# Muskelin regulates actin-dependent synaptic changes and intrinsic brain activity relevant to behavioral and cognitive processes

**DOI:** 10.1038/s42003-022-03446-1

**Published:** 2022-06-15

**Authors:** Mary Muhia, PingAn YuanXiang, Jan Sedlacik, Jürgen R. Schwarz, Frank F. Heisler, Kira V. Gromova, Edda Thies, Petra Breiden, Yvonne Pechmann, Michael R. Kreutz, Matthias Kneussel

**Affiliations:** 1 Institute of Molecular Neurogenetics, Center for Molecular Neurobiology, ZMNH, University Medical Center Hamburg-Eppendorf, Falkenried 94, 20251 Hamburg, Germany; 2 RG Neuroplasticity Leibniz Institute for Neurobiology, 39118 Magdeburg, Germany; 3 Department of Diagnostic and Interventional Neuroradiology, University Medical Center Hamburg-Eppendorf, Hamburg, Germany; 4 Leibniz Group ‘Dendritic Organelles and Synaptic Function’, Center for Molecular Neurobiology, ZMNH, University Medical Center Hamburg-Eppendorf, 20251 Hamburg, Germany; 5 Present Address: Institute of Science and Technology (IST) Austria, Klosterneuburg, Austria; 6 Present Address: Biomedical Engineering Department, Centre for the Developing Brain, School of Biomedical Engineering & Imaging Sciences, King’s College London, London, UK

**Keywords:** Cellular neuroscience, Cognitive neuroscience

## Abstract

Muskelin (Mkln1) is implicated in neuronal function, regulating plasma membrane receptor trafficking. However, its influence on intrinsic brain activity and corresponding behavioral processes remains unclear. Here we show that murine *Mkln1* knockout causes non-habituating locomotor activity, increased exploratory drive, and decreased locomotor response to amphetamine. Muskelin deficiency impairs social novelty detection while promoting the retention of spatial reference memory and fear extinction recall. This is strongly mirrored in either weaker or stronger resting-state functional connectivity between critical circuits mediating locomotor exploration and cognition. We show that *Mkln1* deletion alters dendrite branching and spine structure, coinciding with enhanced AMPAR-mediated synaptic transmission but selective impairment in synaptic potentiation maintenance. We identify muskelin at excitatory synapses and highlight its role in regulating dendritic spine actin stability. Our findings point to aberrant spine actin modulation and changes in glutamatergic synaptic function as critical mechanisms that contribute to the neurobehavioral phenotype arising from *Mkln1* ablation.

## Abstract (web summary)

A murine muskelin knockout induces increased exploratory drive and alters cognition and functional connectivity. These effects correlate with actin-dependent changes in dendritic branching, spine structure, and AMPAR-mediated synaptic transmission.

## Introduction

Muskelin is an intracellular multidomain protein that was first shown to promote cell attachment to the extracellular matrix glycoprotein thrombospondin-1^1^. Subsequent investigations in adherent cell types have implicated muskelin in cytoskeletal organization to influence cell adhesion, morphology, and motile behavior. Suppression of muskelin is shown to alter the dynamics of focal adhesion assembly^1^ and to disrupt stress fiber organization that critically depends on actomyosin contractility^2^. At the same time, muskelin depletion robustly promotes cell migration^2–4^ and results in cells with enlarged perimeters caused by increased ruffling and formation of actin-containing protrusive edges^2,3^.

In neurons, muskelin has a predominantly cytoplasmic distribution, with a punctate expression pattern along axons and dendrites^4,5^. We recently demonstrated that muskelin associates with and modulates the bidirectional transport of cellular prion protein (PrP^C^), a mechanism that contributes to accelerated prion disease onset in *Mkln*1-null mice upon challenge with pathogenic prion^6^. While our previous work showed that muskelin regulates GABA_A_ receptor internalization and trafficking across F-actin and microtubule tracks^7^, it is currently unclear whether muskelin contributes to excitatory synaptic function. Moreover, it is likely that besides its function in regulating receptor turnover at the plasma membrane^7,8^, muskelin may exert a currently unknown role in neuronal actin dynamics, thereby influencing neuronal and synaptic processes that rely on actin remodeling and stability^9,10^.

The function of muskelin in the brain is also largely unexplored despite its expression in brain tissue across development^1,5,11^. Muskelin is enriched in several brain regions, including the cerebral cortex, basal ganglia, cerebellum, and hippocampus^5,12^. Our previous study showed that loss of muskelin induces a robust increase in the power of spontaneously occurring hippocampal sharp-wave ripples (SPW-Rs)^7^, which are shown to play a critical role in memory consolidation^13^. Yet, it is unclear whether the relevance for muskelin may extend to processes in vivo and whether this may depend on its impact at the neuronal network and synaptic levels.

Here, we investigated the relevance of muskelin to behavioral functions and sought to identify corresponding changes in intrinsic brain activity and synaptic correlates of *Mkln1* deletion. We show that *Mkln1*-null mice manifest multiple behavioral alterations and that *Mkln1* deletion can have memory suppressive and promoting effects. The behavioral phenotypes were reflected in altered functional connectivity of resting-state brain networks that underlie locomotor exploration and cognition. In addition, loss of muskelin alters neuronal morphology comprising increased dendritic tree branching and modifications in spine morphology. We identify muskelin localization in excitatory synapses and show that *Mkln1* knockout is associated with enhanced AMPAR-mediated synaptic transmission but impaired capacity of CA1 synapses to maintain LTP following high-frequency stimulation. Our study highlights a role for muskelin in regulating the pool of stable actin in dendritic protrusions, suggesting that aberrant actin cytoskeleton content and stability contributes to the synaptic effects and in vivo phenotypes associated with muskelin dysfunction.

## Results

### *Mkln1*-*null* mice exhibit impaired locomotor habituation to novel environments

**Fig. 1:**
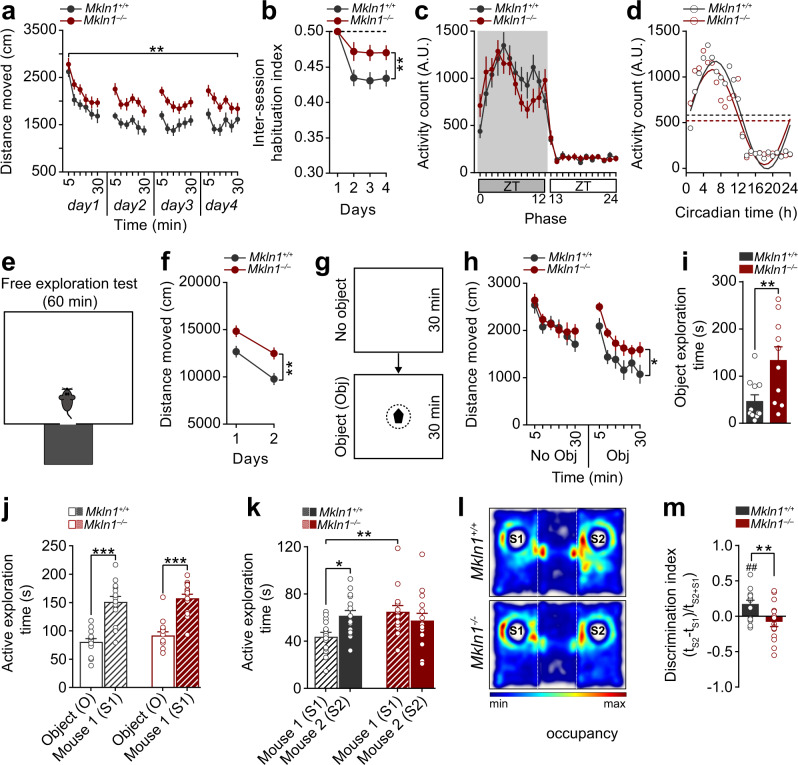
*Mkln1* deletion impairs locomotor habituation and exploratory behavior in novel environments. **a** Assessment of activity in the open-field expressed as a function of 5-min bins revealed sustained locomotion in *Mkln1*^*–/–*^ mice across four test days (genotype: *F*(1,26) = 15.67, *P* < 0.01). **b** An activity change ratio calculated to examine locomotor habituation shows impaired intersession habituation in *Mkln1*^*–/–*^ mice (genotype: *F*(1,26) = 8.0, *P* < 0.01; genotype × days: *F*(3,78) = 3.50, *P* < 0.05). **c** Home cage recordings over three days indicated increased activity levels during the dark (active) phase (shaded area: ZT0-ZT12) and low activity during the light (sleeping) phase of the cycle (ZT13-ZT24) in both genotype groups. **d** Waveform cosine curves (solid lines) with 24 h periods fitted to estimate the circadian rhythm pattern show that *Mkln1* knockout does not alter synchrony to light–dark cycles. Circles correspond to averaged activity at 1-h intervals over three days of recording. Dashed lines on the *y* axis depict an estimate of the central tendency of activity distribution (Midline Estimating Statistic of Rhythm (MESOR)) for each genotype group. **e** Scheme of a modified open-field test in which mice could escape into a dark enclosed compartment. **f** *Mkln1*^*–/–*^ mice displayed pronounced locomotion across 2 test days (genotype: *F*(1,24) = 8.64, *P* < 0.01). **g** Scheme of the open-field experiment in which a novel object was introduced mid-way during the experiment. **h** Both genotype groups showed comparable activity levels during the 30-min (no Obj) session to an empty arena. However, *Mkln1*^*–/–*^ mice displayed a heightened response to the novel object as reflected in significantly increased locomotor activity (genotype × session: *F*(1,17) = 6.21, *P* < 0.05 followed by Bonferroni post hoc test for pairwise comparisons (*P < 0.05)). **i** Time spent actively interacting with the object was significantly higher in *Mkln1*^*–/–*^ mice (genotype: *F*(1,17) = 10.39, *P* < 0.01), indicating enhanced exploration. **j** In the three-chamber social interaction test, both genotypes demonstrate clear sociability by spending significantly more time interacting with a novel conspecific (S1) over an inanimate object (O) with no social valence (stimulus type: *F*(1,26) =  108.45, *P* < 0.0001). **k** Deficient preference for social novelty in *Mkln1*^*–/–*^ mice during the recall session. Compared with *Mkln1*^*+/+*^ mice, *Mkln1*^*–/–*^ mice spent significantly more time in contact with the now-familiar (S1) conspecific. Also, *Mkln1*^*–/–*^ mice engaged with the S1 and S2 conspecifics equally (social stimulus × genotype: *F*(1,26) = 6.11, *P* < 0.05) followed by Bonferroni post hoc test for pairwise comparisons (*P < 0.05, **P < 0.01). **l** Representative heat map images depicting the animal’s nose-point cumulative location within each compartment during the novelty preference (recall) session. A high signal (occupancy) is detected around the active exploration area. **m** The discrimination index was negative and significantly lower in *Mkln1*^*–/–*^ mice relative to *Mkln1*^*+/+*^ controls (genotype: *F*(1,26) = 8.25, *P* < 0.01), indicating impaired social memory. Only *Mkln1*^*+/+*^ controls show a preference for the novel conspecific that differs significantly from random exploration (one-sample *t*-test: ^##^*P* < 0.01). For data related to (**a**, **b**) (*Mkln1*^*+/+*^ (*N* = 15: *M* = 8, *F* = 7))*; Mkln1*^*–/–*^ (*N* = 15: *M* = 8, *F* = 7); **c**, **d** (*Mkln1*^*+/+*^ (*N* = 22: *M* = 11, *F* = 11)*; Mkln1*^*–/–*^ (*N* = 18: *M* = 11, *F* = 7); **e**, **f** and **j**–**m** (*Mkln1*^*+/+*^ (*N* = 15: *M* = 8, *F* = 7)*; Mkln1*^*–/–*^ (*N* = 15: *M* = 8, *F* = 7); **g**–**i** *Mkln1*^*+/+*^ (*N* = 11: *M* = 5, *F* = 6)*; Mkln1*^*–/–*^ (*N* = 10: *M* = 5, *F* = 5). Data are presented as means ± SEM.

Given that little is known about the relevance of muskelin in vivo, we tested *Mkln1*-null mice and control littermates in a series of behavioral experiments designed to assess exploratory activity, anxiety-related behavior, and cognitive abilities. Two independent factors (genotype and sex) were included in the statistical analyses to determine whether the behavioral outcomes are altered by genotype, sex, or the interaction of both factors. A summary of statistical analyses for the behavioral tests is provided in Supplementary Table 1. Initial assessment in the open-field assay revealed an apparent genotype effect in the activity profile, with *Mkln1*^*–/–*^ mice, but not *Mkln1*^*+/–*^ mice, demonstrating a significant increase in locomotor activity over a 4-day testing period relative to *Mkln1*^*+/+*^ controls (Fig. 1a and Supplementary Fig. 1). While both genotype groups showed comparable within-session habituation (Supplementary Fig. 2a), *Mkln1*^*–/–*^ mice failed to habituate to the same extent as *Mkln1*^*+/+*^ controls in response to 24 h temporal intervals (Fig. 1b). Previous evidence indicates that locomotor habituation may be influenced by changes in anxiety responses, with more anxious mice shown to increase interday activity levels as they become less anxious^14^. Here, it is unlikely that impaired habituation in *Mkln1*^*–/–*^ mice stemmed from differences in anxiety-related behavior since the fraction of time spent in the open-field center was comparable between genotypes (Supplementary Fig. 2b). Further testing in the elevated plus maze and light–dark transition tests confirmed intact basal anxiety levels in *Mkln1*^*–/–*^ mice (Supplementary Fig. 2c, d).

To confirm whether *Mkln1*^*–/–*^ mice were constitutively more active than controls, we monitored home cage activity levels across the light–dark cycle for three consecutive days. Mice from both genotype groups demonstrated increased activity levels in the dark (active) phase and decreased activity levels in the light (sleeping) phase that is characteristic of a typical circadian activity profile (Fig. 1c). We found that male *Mkln1*^*+/+*^ controls showed lower activity levels relative to female *Mkln1*^*+/+*^ mice during the dark phase (Supplementary Table 1); however, there were no genotype differences in overall activity levels during both stages of the cycle (Supplementary Fig. 2e), indicating that *Mkln1*^*–/–*^ mice do not suffer from general hyperactivity. Waveform cosine curves with 24 h periods fitted to estimate circadian rhythm pattern showed that *Mkln1* knockout does not alter entrainment to a 12 h:12 h light–dark cycle (Fig. 1d). Therefore, we focused on whether the locomotor deficits in *Mkln1*^*–/–*^ mice might instead reflect impaired ability to adapt to novelty or abnormal exploratory activity.

### *Mkln1-null* mice exhibit increased exploratory drive and enhanced novelty-seeking behavior

In rodents, the pattern of exploratory behavior appears to depend on the conditions under which it occurs; rats that are free to explore a novel environment display a different pattern of exploration than those forced to explore an enclosed open field^15,16^. In the open-field assay described above (Fig. 1a), we observed an increased incidence of stereotypical jumping in arena corners during testing, which is often associated with escaping attempts from enclosed novel environments^15^. Therefore, we examined exploratory activity using an open field in which mice could explore the open arena but were also allowed to escape into a dark enclosed compartment (Fig. 1e). This approach affords the evaluation of exploratory behavior independent of confounds arising from escaping attempts in confined spaces. Having adjusted for time spent in the open arena, *Mkln1*^*–/–*^ mice again covered greater distances than controls, which remained significantly elevated by the second day of testing (Fig. 1f).

Next, mice were tested for responses to a change in environmental properties by introducing an unfamiliar object mid-way during an open-field experiment (Fig. 1g). Renewed exploratory activity occurs following the introduction of a novel object in a familiar environment^17^, which can also assess conflict in approach-avoidance behavior incurred by a potentially dangerous object^18^. Although locomotion was initially comparable between genotypes (no Obj), the presence of the novel object elicited a pronounced increase in activity levels in *Mkln1*^*–/–*^ mice compared with *Mkln1*^*+/+*^ controls (Obj; Fig. 1h). The initial response induced by the novel object revealed a marginally significant reduction in the time taken to approach the object in *Mkln1*^*–/–*^ mice (Supplementary Fig. 2f); the lower neophobic reaction to novelty might reflect decreased perception of risk^18^ or increased novelty-seeking behavior. We observed a significant increase in the overall time spent investigating the object (Fig. 1i), consistent with the interpretation that *Mkln1* knockout alters exploratory behavior.

### *Mkln1-null* mice display clear sociability but impaired preference for social novelty

To further determine the impact of *Mkln1* deletion on exploration and response to novelty, we investigated *Mkln1*^*–/–*^ mice for sociability and the ability to detect social novelty in the three-chamber social interaction paradigm^19^. There was no genotype effect on time spent in each compartment during a 10-min habituation session (Supplementary Fig. 2g). Likewise, both genotype groups preferred interacting with a novel conspecific over an inanimate object (Fig. 1j), indicating that *Mkln1* deletion does not alter social-seeking behavior. However, a clear difference emerged when mice were tested for social recognition in the subsequent recall session. While *Mkln1*^*+/+*^ mice spent significantly more time in contact with a novel mouse (S2) over the previous familiar conspecific (S1), *Mkln1*^*–/–*^ mice failed to show an appreciation of social novelty by engaging with both social stimuli to similar extents (Fig.1k–l). We found that *Mkln1*^*–/–*^ mice also showed a discrimination performance below zero (Fig. 1m) owing to renewed interest in the familiar mouse (S1) during the recall session (Fig. 1k). Hence, although the baseline social approach is mainly intact, *Mkln1*^*–/–*^ mice display impaired social recognition as indicated by deficient habituation to a novel conspecific.

### *Mkln1-null* mice exhibit decreased locomotor sensitivity to amphetamine but intact sensorimotor gating function

**Fig. 2:**
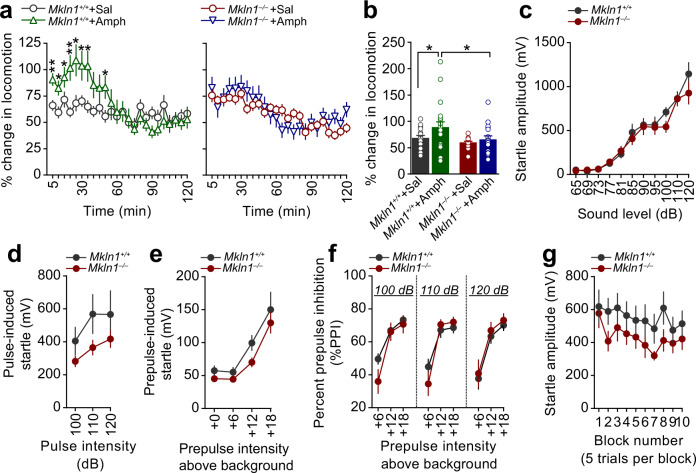
*Mkln1-null* mice exhibit intact sensorimotor gating function but decreased locomotor sensitivity to amphetamine. **a** Locomotor response to acute amphetamine (2.5 mg/kg) challenge after considering differences in baseline locomotion. Compared with the respective saline-treated groups, amphetamine significantly increased locomotor activity in *Mkln1*^*+/+*^ mice (left panel) but had negligible effect in *Mkln1*^*–/–*^ mice (right panel; genotype × drug × time bins: (*F*(23,1518) = 2.23, *P* < 0.01). Restricted analyses to each genotype groups revealed a pronounced drug effect in *Mkln1*^*+/+*^ mice (drug × time bins: *F*(23,782) = 3.20, *P* < 0.0001) followed by Bonferroni post hoc test for pairwise comparisons (*P < 0.05, **P < 0.01) but not in the *Mkln1*^*–/–*^ group. **b** Overall, *Mkln1*^*–/–*^ mice showed significantly diminished locomotor response to amphetamine compared with *Mkln1*^*+/+*^ controls (genotype: *F*(1,66) = 5.47, *P* < 0.05) followed by Bonferroni post hoc test for pairwise comparisons (*P < 0.05). **c** Both *Mkln1*^*–/–*^ and *Mkln1*^*+/+*^ mouse groups show a comparable increase in startle reactivity in response to pulse stimuli of increasing sound intensity (dB intensity: *F*(9,144) = 65.82, *P* < 0.0001). **d**, **e** The absence of muskelin did not affect startle response to pulse-alone (**d**) and prepulse-alone (**e**) stimuli. **f** Percent PPI depicted as function of increasing prepulse intensities for each pulse intensity (prepulse intensities: +6, +12, and +18 dB above background (65 dB) noise). *Mkln1*^*–/–*^ mice show highly similar PPI levels as *Mkln1*^*+/+*^ controls (prepulse: *F*(2,52) = 149.17, *P* < 0.0001). **g** Habituation of startle responses following the presentation of 120 dB acoustic stimuli. Each block comprises 5 trials of acoustic stimuli. Both genotypes show a decline in the response amplitude as a function of trials (trial: *F*(9,153) = 3.14, *P* < 0.01). For data related to **a**, **b** (Amph: *Mkln1*^*+/+*^ (*N* = 21: *M* = 10, *F* = 11)*; Mkln1*^*–/–*^ (*N* = 18: *M* = 7, *F* = 11); Saline: *Mkln1*^*+/+*^ (*N* = 17: *M* = 8, *F* = 9)*; Mkln1*^*–/–*^ (*N* = 18: *M* = 8, *F* = 10); **c** (*Mkln1*^*+/+*^ (*N* = 10: *M* = 5, *F* = 5)*; Mkln1*^*–/–*^ (*N* = 10: *M* = 5, *F* = 5); **d**–**f** (*Mkln1*^*+/+*^ (*N* = 15: *M* = 8, *F* = 7)*; Mkln1*^*–/–*^ (*N* = 15: *M* = 8, *F* = 7); **g** (*Mkln1*^*+/+*^ (*N* = 11: *M* = 5, *F* = 6)*; Mkln1*^*–/–*^ (*N* = 10: *M* = 5, *F* = 5). Data are presented as means ± SEM.

Given our observations above, we investigated whether *Mkln1*^*–/–*^ mice manifest other behavioral alterations relevant to hyperactivity-related disorders such as schizophrenia (SCZ), attention deficit hyperactivity disorder (ADHD), and mania in bipolar disorder^20^. We first investigated locomotor responses to the psychostimulant amphetamine, which is shown to exert distinct effects in mouse models of the three disorders^20^. During baseline recording in the open field, *Mkln1*^*–/–*^ mice showed apparent differences in locomotor activity relative to *Mkln1*^*+/+*^ controls (Supplementary Fig. 3a). Since pre-drug differences in activity levels may account for differential responses to amphetamine, post-injection locomotor responses for each mouse were normalized to baseline locomotion. As expected, amphetamine (2.5 mg/kg, i.p.) led to pronounced locomotion in *Mkln1*^*+/+*^ mice, which persisted for 30 min compared with the corresponding saline-injected group (left panel, Fig. 2a). By contrast, amphetamine failed to augment locomotor activity in *Mkln1*^*–/–*^ mice relative to their respective saline-treated group (right panel, Fig. 2a). Interestingly, the locomotor profile of saline-treated *Mkln1*^*–/–*^ mice matched that of amphetamine-treated *Mkln1*^*+/+*^ mice (Supplementary Fig. 3a), suggesting a ceiling effect due to elevated baseline activity levels. This possibility is reflected in the significantly blunted response to amphetamine in *Mkln1*^*–/–*^ mice compared with *Mkln1*^*+/+*^ controls (Fig. 2b).

Next, we tested prepulse inhibition (PPI) and habituation of the acoustic startle response; two distinct behavioral measures of central information-processing mechanisms, and startle plasticity^21,22^. Initial screening for acoustic startle responses revealed comparable startle reactivity between genotypes (Fig. 2c), suggesting intact auditory acuity and basal acoustic startle responses in *Mkln1*^*–/–*^ mice. In the PPI protocol employed here^23^, *Mkln1*^*–/–*^ mice startled to a similar degree as *Mkln1*^*+/+*^ mice in response to pulse-alone and prepulse-only trials (Fig. 2d, e). Furthermore, *Mkln1*^*–/–*^ and *Mkln1*^*+/+*^ mice showed the expected PPI pattern, as indicated by the increase in the magnitude of PPI (%) with increasing prepulse intensity (Fig. 2f). Overall percentage PPI was comparable between genotype groups (*Mkln1*^*+/+*^ mice: 60.20% ± 3.04; *Mkln1*^*–/–*^ mice: 59.0% ± 3.75), indicating that *Mkln1* knockout does not alter sensorimotor gating function. An acoustic startle habituation procedure was used to assay sensorimotor adaptation and allowed us to differentiate the impact of *Mkln1* deletion on habituation of motivated behavior (locomotion) versus evoked (startle) behavior^24^. Short-term habituation of the startle reflex was normal in *Mkln1*^*–/–*^ mice, with both genotypes demonstrating a similar response amplitude following the repeated presentation of white-noise pulses of 120 dB intensity (Fig. 2g).

### Enhanced spatial reference memory and fear-extinction recall in *Mkln1-null* mice

**Fig. 3:**
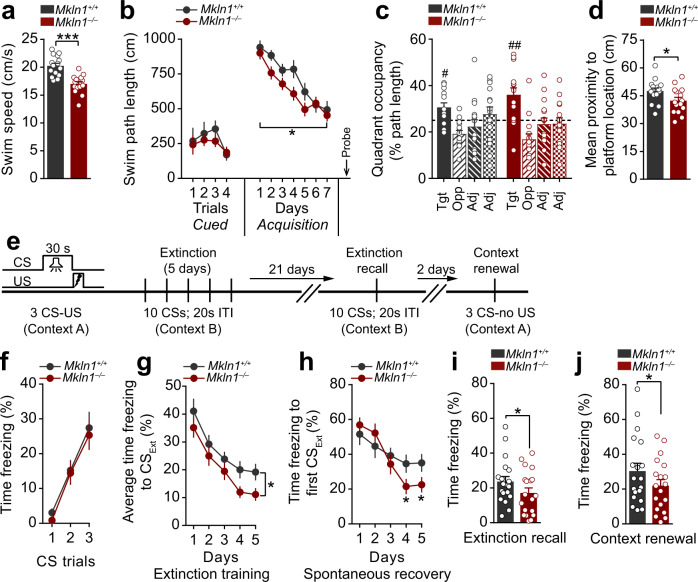
*Mkln1-null* mice exhibit enhanced spatial reference memory retention and greater fear extinction recall. **a** Significantly lower swim speed in *Mkln1*^*–/–*^ mice during water maze training (genotype: *F*(1,26) = 24.91, *P* < 0.0001). **b** Mice from both genotype groups demonstrate equal distance (path length) to reach a visible (Cued) escape platform. Training in the hidden platform task (Acquisition) to assess spatial reference memory revealed clear learning across 7 days of training in both genotype groups (days: *F*(6,156) = 20.94, *P* < 0.0001). However, swim path length was significantly shorter in *Mkln1*^*–/–*^ mice compared with the *Mkln1*^*+/+*^ control group (genotype: *F*(1,26) = 6.97, *P* < 0.05). **c** The proportion of swim track (% path length in individual quadrants during the probe trial. Both genotype groups showed significant spatial bias for the target quadrant (quadrant: *F*(3,78) = 8.86, *P* < 0.0001) that different significantly from chance as depicted by the dashed line (one-sample t-test against 25% random search: ^#^*P* < 0.05, ^##^*P* < 0.01). **d** Proximity to the escape platform throughout the probe trial. This is indexed as the mean cumulative distance between the mouse and target center, which was significantly shorter for *Mkln1*^*–/–*^ mice relative to *Mkln1*^*+/+*^ controls (genotype: *F*(1,26) = 5.87, *P* < 0.05). **e** Scheme depicting the cued fear conditioning paradigm to examine associative fear learning and extinction of fear memories. Fear acquisition in context A; extinction training and recall in a novel and neutral context B; test for fear renewal in context A. **f** Both genotypes show equivalent freezing levels to the discrete CS-tone during conditioned fear acquisition. **g** Percent time freezing to non-reinforced trials during extinction training. *Mkln1*^*–/–*^ mice showed significantly lower freezing responses across five extinction sessions compared with *Mkln1*^*+/+*^ mice (genotype: *F*(1,34) = 5.27, *P* < 0.05). **h** Assessment of freezing responses at the outset of each extinction session (first CS trial) shows significant attenuation of spontaneous fear recovery in *Mkln1*^*–/–*^ mice with continued extinction training (days × genotype: *F*(4,136) = 4.28, *P* < 0.01) followed by Bonferroni post hoc test for pairwise comparisons (*P < 0.05). **i** Enhanced long-term extinction memory recall in *Mkln1*^*–/–*^ mice compared with *Mkln1*^*+/+*^ mice (genotype: *F*(1,34) = 5.29, *P* < 0.05). **j** Return of fear through contextual renewal was significantly lower in *Mkln1*^*–/–*^ mice (genotype: *F*(1,34) = 4.54, *P* < 0.05). Data related to (**a**–**d**) (*Mkln1*^*+/+*^ (*N* = 15: *M* = 8, *F* = 7)*; Mkln1*^*–/–*^ (*N* = 15: *M* = 8, *F* = 7); **e**–**j** (*Mkln1*^*+/+*^ (*N* = 19: *M* = 8, *F* = 11)*; Mkln1*^*–/–*^ (*N* = 19: *M* = 7, *F* = 12). Data are presented as means ± SEM.

Considering that diminished preference for social novelty (Fig. 1k) may reflect a general cognitive deficit in *Mkln1*^*–/–*^ mice, we extended our evaluation to address the contribution of muskelin to other forms of learning and memory. Assessment for spatial reference memory in the water maze revealed significant swim speed differences between *Mkln1*^*–/–*^ and *Mkln1*^*+/+*^ mice during training (Fig. 3a). Hence, distance to navigate to the platform (path length) was analyzed as the dependent variable as this measure is free from potential confounds in swim speed^25^. Mice from each genotype group performed comparably in the visible platform (non-spatial) version of the task (Cued; Fig. 3b), demonstrating adequate swimming abilities and motivation to escape the water in *Mkln1*^*–/–*^ mice. When assessed for spatial navigation abilities, both genotype groups showed a decline in path length across training. Although the rate of spatial learning did not differ between genotypes, there was a significant main effect of genotype, such that overall task performance was better in *Mkln1*^*–/–*^ mice than *Mkln1*^*+/+*^ controls (Acquisition; Fig. 3b). Testing in the subsequent probe trial revealed a clear preference for the training quadrant in both genotype groups, although *Mkln1*^*–/–*^ mice showed a more robust spatial bias for the target quadrant (Fig. 3c). Furthermore, the mean search error, a proximity measure shown to be a more reliable index of search accuracy^26^, was significantly lower in *Mkln1*^*–/–*^ mice (Fig. 3d), suggesting enhanced spatial memory retention following *Mkln1* knockout.

We next sought to address whether muskelin modulates the acquisition and expression of fear memories using auditory cued fear conditioning (Fig. 3e). Mice from both genotype groups showed a comparably rapid increase in freezing levels during conditioned acquisition (Fig. 3f). Subsequent extinction training in a distinct neutral context revealed that non-reinforced CS exposure was sufficient to induce a rapid decline of the conditioned fear response in both genotype groups (Fig. 3g). However, *Mkln1*^*–/–*^ mice froze significantly less than *Mkln1*^*+/+*^ mice (Fig. 3g) and showed decreased spontaneous fear recovery (Fig. 3h) during extinction training. It is reported that the magnitude of spontaneous fear recovery is dependent on the delay interval between two extinction sessions; the longer the retention interval between sessions, the higher the recovery^27^. Therefore, mice underwent an extinction retest phase 21 days later, which revealed the persistency of the extinction memory and diminished spontaneous fear recovery in *Mkln1*^*–/–*^ mice (Fig. 3i). Fear attenuation was long-lasting in *Mkln1*^*–/–*^ mice, which showed a significant reduction of freezing responses upon re-introduction to the original training context (Fig. 3j). These observations suggest that while *Mkln1* deletion may impair certain forms of cognition such as social memory (Fig. 1m), it leads to robust spatial memory retention and promotes the expression of fear-extinction memories.

### Widespread alterations in resting-state functional connectivity but intact brain tissue microstructure in *Mkln1*-null mice

**Fig. 4:**
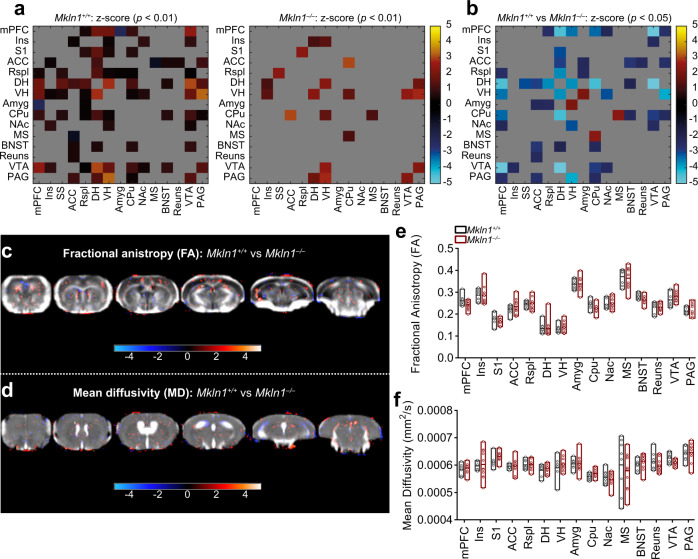
Widespread alterations in resting-state functional connectivity but largely intact brain tissue microstructure in *Mkln1-null* mice. **a**, **b** Resting-state functional connectivity (FC) analyses of BOLD signal synchronization of fifteen regions of interest (ROI). **a** Connectivity matrices of *Mkln1*^*+/+*^ mice (left panel) and *Mkln1*^*–/–*^ mice (right panel), in which functional correlations (*z*-score) between pairs of regions are depicted by color scale. Compared with *Mkln1*^*+/+*^ control mice, *Mkln1*^*–/–*^ mice displayed visibly weaker interregional FC. **b** Statistical analyses of FC strength are depicted as a matrix, revealing the direction of significant genotype differences for each ROI-ROI connection. Relative to *Mkln1*^*+/+*^ control mice, blue-to-light blue elements indicate regions of significantly reduced FC, while red elements depict regions exhibiting significantly increased FC in *Mkln1*^*–/–*^ mice. **c**–**f** Diffusion tensor imaging (DTI) in *Mkln1*^*+/+*^ and *Mkln1*^*–/–*^ mice. Diffusion parameters (fractional anisotropy (FA) and mean diffusivity (MD) were analyzed using an ROI-based approach. (**c**, **d**) Representative set of six axial FA and MD images. The FA (**c**) and MD (**d**) maps are overlaid with resulting significant *t* values (*P* < 0.05) following a voxel-wise *t*-test comparing the DTI data between the two genotype groups. Also shown are the *z*-score values in a color scale ranging from −4 to 4. **e**, **f** Bar graphs illustrate genotype-dependent comparisons of FA and MD values in the different ROIs. Values represent mean ± SEM. (*Mkln1*^*+/+*^ (*N* = 8)*; Mkln1*^*–/–*^ (*N* = 11). Brain region abbreviations are defined in Supplementary Fig. S2.

One explanation for the neural basis of the behavioral outcomes is modifications in the structural or functional connectivity patterns among circuits that govern different cognitive and noncognitive processes^28,29^. Therefore, we evaluated the consequence of *Mkln1* knockout on intrinsic functional connectivity mapping using resting-state functional magnetic resonance imaging (rsfMRI). Resting-state fMRI examines temporal correlations of blood oxygen level-dependent (BOLD) fluctuations across brain regions at rest^30^, which can reflect spontaneous neuronal synchronization of the brain^31^. Analysis of BOLD signal synchronization revealed widespread interregional correlations in *Mkln1*^*+/+*^ mice (Fig. 4a, left panel), but significant alterations in resting-state functional connectivity in *Mkln1*^*–/–*^ mice (Fig. 4a, right panel). In particular, *Mkln1*^*–/–*^ mice were characterized by functional decoupling that mainly affected connections between the dorsal hippocampus (DH), anterior cingulate cortex (ACC), and medial prefrontal cortex (mPFC) (Fig. 4b). At the same time, we found significantly increased synchronizations between the ventral hippocampus (VH) and the amygdala (Amy) and between the caudate–putamen (CPu) and medial septum (MS) in *Mkln1*^*–/–*^ mice (Fig. 4b). Further analyses of homotopic interhemispheric connectivity indicated that *Mkln1*^*–/–*^ mice generally lack functional synchronization with the contralateral side (Supplementary Fig. 3b).

Given that the neural microstructure state may influence functional coupling, we investigated brain water diffusivity with diffusion tensor imaging (DTI), which permits identification of white and gray matter integrity. Assessment of diffusion tensor derived indices (fractional anisotropy, FA and mean diffusivity, MD) yielded significantly reduced FA in the right CPu (Supplementary Fig. 3c), suggesting diminished structural organization of fiber tracts in this region. However, no significant genotype-dependent differences were detected on both measures in other regions (Fig. 4c–f and Supplementary Fig. 3c, d), indicating that microstructural integrity is mainly intact in *Mkln1*^*–/–*^ mouse brains. These findings suggest that *Mkln1* genetic ablation reshapes bilateral and homotopic functional connectivity without inducing neural microstructural damage.

### Loss of *Mkln1* leads to increased dendritic tree branching and altered dendritic spine morphology

**Fig. 5:**
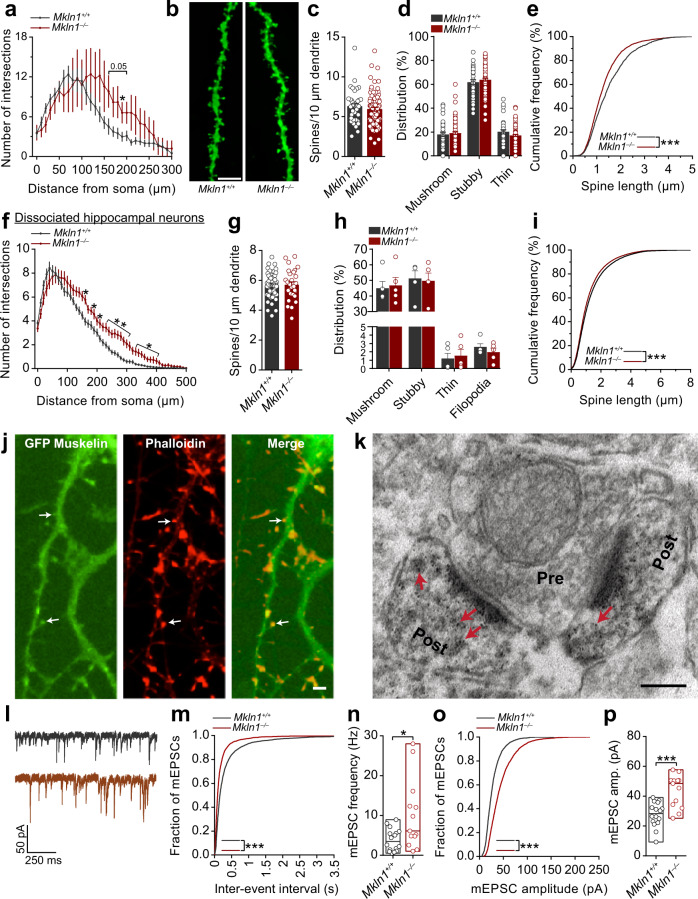
*Mkln1* deletion alters neuronal morphology and enhances AMPAR-mediated synaptic transmission. **a** Sholl analysis of the dendritic tree architecture revealed a strong trend for increased dendrite complexity in CA1 pyramidal neurons of *Mkln1*^*–/–*^ mice (genotype × radius: *F*(30,330) = 1.65, *P* < 0.05 followed by Bonferroni post hoc test for pairwise comparisons (*P* = 0.05, *P < 0.05). *Mkln1*^*+/+*^ (*n* = 8 neurons) and *Mkln1*^*–/–*^ (*n* = 5 neurons). **b** Representative images of DIL dye staining of a dendrite segment and spines from *Mkln1*^*–/–*^ and *Mkln1*^*+/+*^ CA1 pyramidal neurons. Scale bar = 5 µm. **c** Graphical depiction of comparable spine density in apical branches from *Mkln1*^*–/–*^ and *Mkln1*^*+/+*^ CA1 pyramidal neurons (genotype: *β* = −0.29, *t* = −0.72, *P* = 0.47). **d** Spine ty*p*e distribution on apical branches was identical for *Mkln1*^*–/–*^ and *Mkln1*^*+/+*^ CA1 pyramidal neurons. *Mkln1*^*+/+*^ (*n* = 11 neurons, 37 dendrites), *Mkln1*^*–/–*^ (*n* = 35 neurons, 86 dendrites). The mixed model analysis accounts for non-independence in the data from multiple dendrites per neuron. Data are shown as mean ± SEM. **e** Spine lengths of all analyzed spines plotted as a cumulative frequency, which reveals a significant shift towards shorter spine lengths in *Mkln1*^*–/–*^ CA1 pyramidal neurons (two-sample Kolmogorov–Smirnov (KS) test: *D* = 0.126, *P* < 0.0001). *Mkln1*^*+/+*^ (*n* = 66 dendrites, 2202 spines), *Mkln1*^*–/–*^ (*n* = 146 dendrites, 4592 spines). Data (**a**–**e**) are derived from three mice per genotype. **f** Sholl analysis of the dendritic arbor shows that *Mkln1* deletion significantly increases dendritic branching in dissociated hippocampal neurons (Genotype: *F*(1,19) = 8.53, *P* < 0.01); genotype × radius: *F*(49,931) = 2.67, *P* < 0.0001) followed by Bonferroni post hoc test for pairwise comparisons (*P < 0.05, **P < 0.01). *Mkln1*^*+/+*^ (116 neurons from *n* = 12 embryos) and *Mkln1*^*–/–*^ (90 neurons from *n* = 9 embryos). Analysis treating each embryo (instead of each neuron) as an independent sample. Spine density (**g**) and ratio of spine types (**h**) was comparable between *Mkln1*^*+/+*^ and *Mkln1*^*–/–*^ cultured hippocampal neurons (Genotype: *β* = −0.03, *t* = −0.12, *P* = 0.9). *Mkln1*^*+/+*^ (*n* = 55 neurons) and *Mkln1*^*–/–*^ (*n* = 28 neurons). Data are shown as mean ± SEM. **i** The cumulative frequency distribution of spine length shows a significant leftward shift (shorter spines) for *Mkln1*^*+/+*^ spines (two-sample KS test: *D* = 0.049, *P* < 0.0001). *Mkln1*^*+/+*^*:* (*n* = 55 neurons, 73,422 spines), *Mkln1*^*–/–*^: (*n* = 28 neurons, 39733 spines). Data (**f**–**i**) are derived from 5 independent preparations. **j** Cultured hippocampal neurons expressing GFP-tagged muskelin (green: left panel) and incubated with fluorochrome-labeled phalloidin (red: middle panel). Arrowheads in the merged right panel point to muskelin localization in a subset of actin-rich dendritic protrusions. Scale bar, 5 µm. **k** Electron micrograph showing immunogold labeling of mouse hippocampal ultrasections with antibodies against muskelin. Muskelin signals are detected in dendritic spines (Post, arrowheads) opposite an axon terminal (Pre). Scale bar, 150 nm. **l**–**p** *Mkln1* deletion leads to enhanced AMPAR-mediated excitatory synaptic transmission. **l** Sample traces from whole-cell voltage-clamp recordings with downward deflections indicating AMPA-mediated mEPSCs in *Mkln1*^*+/+*^ (top) and *Mkln1*^*–/–*^ (bottom) neurons. **m** Cumulative probability plots showing shorter interevent intervals in *Mkln1*^*–/–*^ neurons (two-sample KS test: *D* = 0.23, *P* < 0.0001). **n** AMPA-mEPSC burst frequency was significantly increased in *Mkln1*^*–/–*^ neurons (Mann–Whitney *U* test: *U* = 56.5, *P* < 0.05). **o** Cumulative distribution plot reveals a significant shift towards larger mEPSC amplitudes in *Mkln1*^*–/–*^ neurons (two-sample KS test: *D* = 0.33, *P* < 0.0001). **p** Graphical representation of mean mEPSC amplitudes per neuron, which were significantly increased in *Mkln1*^*–/–*^ neurons (Mann–Whitney *U* test: *U* = 29, *P* < 0.0001). Floating bars represent the min-to-max values. Scatter plots depict results per neuron, and the line within bar graphs corresponds to the group median value. *Mkln1*^*+/+*^ (*n* = 16 neurons); *Mkln1*^*–/–*^ (*n* = 13 neurons) from three independent preparations.

Neural structural connectivity and coupling strength between neuronal clusters may influence the degree of correlated and anti-correlated activity between nodes^32^. Thus, the effect of *Mkln1* deletion on neuronal architecture, including structural or functional modifications at the synapse level, may affect local neuronal network connectivity, influencing activity fluctuations between functionally connected areas of the brain. To address these possibilities, we focused on hippocampal neuronal morphology due to the significantly altered BOLD signal correlation between the hippocampus and other regions (Fig. 4a, b and Supplementary Fig. 3b) and given that muskelin is highly expressed in the hippocampus^5^. Morphometric analysis in fixed brain tissue revealed modifications in the dendritic architecture of *Mkln1*^*–/–*^ CA1 pyramidal neurons, corresponding to increased branching relative to *Mkln1*^*+/+*^ controls (Fig. 5a). Analysis of the number of dendritic spines (Fig. 5b) as a correlate of excitatory synapses revealed comparable spine density (Fig. 5c) and spine type distribution (Fig. 5d) in apical branches of *Mkln1*^*–/–*^ and *Mkln1*^*+/+*^ neurons. Similarly, spine density in basal branches was comparable between *Mkln1*^*–/–*^ and *Mkln1*^*+/+*^ neurons (Supplementary Fig. 4a), although we observed a significant increase in mushroom-type spines and decreased stubby spines along basal branches (Supplementary Fig. 4b). Additional assessment to characterize spine morphology revealed shorter dendritic spines in *Mkln1*^*–/–*^ neurons, as indicated in the significant leftward shift in the cumulative distribution curve (Fig. 5e). At the same time, *Mkln1*^*–/–*^ spines showed a rightward shift in the distribution of spine head diameters (Supplementary Fig. 4c).

Further evaluation in dissociated hippocampal neurons confirmed that *Mkln1* deletion significantly alters dendritic tree ramification. We observed a significant increase in dendritic branch crossings with increasing distance from the cell body of *Mkln1*^*–/–*^ neurons (Fig. 5f). Similar to observations in CA1 pyramidal neurons, spine density and distribution of spine types were unchanged in *Mkln1*^*–/–*^ neurons compared with *Mkln1*^*+/+*^ controls (Fig. 5g, h), indicating that muskelin depletion has minimal impact on dendritic spine numbers. Again, *Mkln1*^*–/–*^ spines showed a rightward shift towards larger spine head diameters (Supplementary Fig. 4d) and a significant shift in spine lengths towards smaller values (Fig. 5i) relative to *Mkln1*^*+/+*^ spines. These findings indicate that the loss of muskelin leads to a more complex dendritic tree arborization in hippocampal neurons. Moreover, *Mkln1* deletion alters the dendritic spine character, leading to wider spine heads but shorter spines.

### Muskelin is localized at excitatory synapses and regulates AMPAR-mediated synaptic transmission

Given that most dendritic spine heads receive excitatory input^33^, the above alterations in spine morphology prompted us to examine whether muskelin may be localized in excitatory synapses. Visualization of GFP-tagged muskelin in primary hippocampal neurons revealed muskelin distribution along dendritic shafts but also localization in a subset of actin-rich dendritic protrusions (Fig. 5j). Immunogold labeling and ultrastructural assessment using high-resolution electron microscopy confirmed muskelin expression in asymmetric synapses, as indicated by the localization of muskelin-positive signals in spines apposed to presynaptic terminals (Fig. 5k).

Next, we assessed whether muskelin is involved in excitatory synaptic transmission and whether changes in functional synaptic mechanisms may contribute to the in vivo phenotypes. We performed whole-cell path-clamp recordings from cultured hippocampal neurons (*DIV* 20–21) derived from *Mkln1*^*+/+*^ and *Mkln1*^*–/–*^ mice. After adjustment of resting potential to −60 mV, action potential (AP) recordings revealed comparable threshold potential (*Mkln1*^*+/+*^ (*n* = 12): 12.98 ± 0.62 mV; *Mkln1*^*–/–*^ (*n* = 14):15.21 ± 0.99 mV) and action potential amplitude (*Mkln1*^*+/+*^ (*n* = 12): 86.08 ± 2.3 mV; *Mkln1*^*–/–*^ (*n* = 14): 78.71 ± 3.2 mV) in neurons from both genotype groups. A similar fraction of neurons from each genotype group showed spontaneous activity, indicating that neurons were excitable and healthy. After isolating AMPAR-mediated miniature excitatory postsynaptic currents (mEPSCs; Fig. 5l), voltage-clamp recordings revealed a shift in interevent intervals toward smaller values in *Mkln1*^*–/–*^ neurons (Fig. 5m). The concomitant increase in event frequency (Fig. 5n) suggested that the absence of muskelin alters presynaptic release probability as opposed to increasing synapse density (see Fig. 5c, g). We also detected a dramatic rightward shift in mEPSC amplitudes towards larger values (Fig. 5o, p), indicating that *Mkln1* knockout induces, at the same time, a postsynaptic effect in terms of receptor abundance at synaptic sites. These observations suggest that in addition to our previous findings at GABAergic synapses^7^, muskelin plays a critical role in glutamatergic synaptic function.

### *Mkln1* deletion impairs high-frequency-mediated long-term synaptic strengthening

**Fig. 6:**
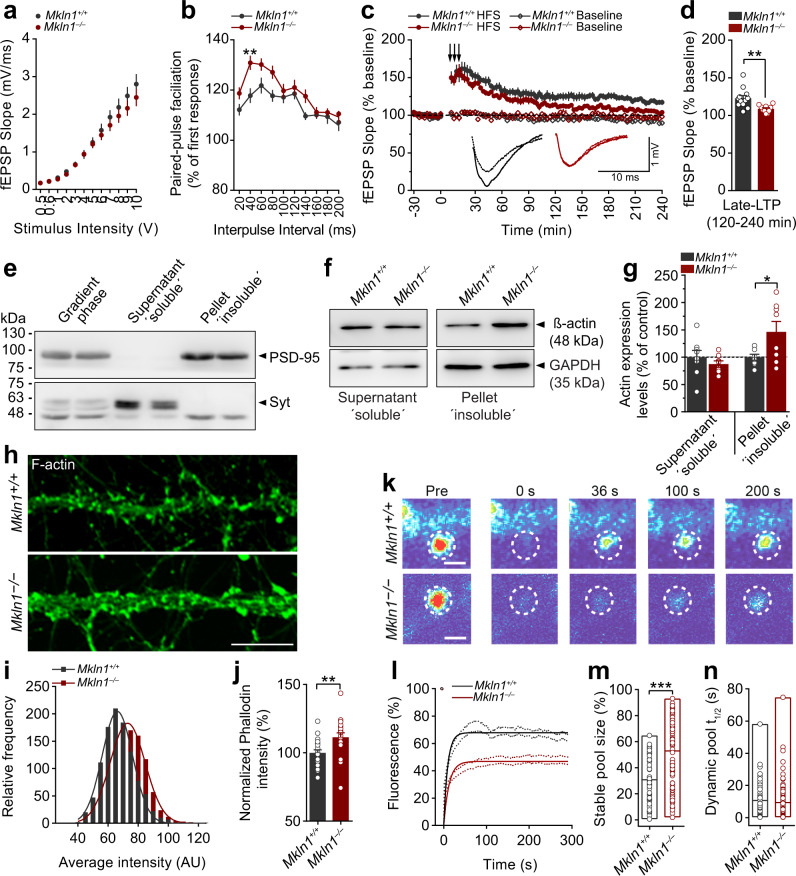
*Mkln1* deletion impairs long-term synaptic potentiation and leads to modifications in spine actin. **a**, **b** Basal synaptic transmission in acute hippocampal slices. **a** Input–output (I/O) curves obtained by plotting the field excitatory postsynaptic potentiation (fEPSP) slope against stimulus intensity were comparable between *Mkln1*^*–/–*^ and *Mkln1*^*+/+*^ slices. **b** The paired-pulse ratio was recorded across different interstimulus intervals (ISI). Note the significantly larger paired-pulse facilitation (PPF) in *Mkln1*^*–/–*^ slices relative to *Mkln1*^*+/+*^ slices (genotype: *F*(1,31) = 12.31, *P* < 0.01). PPF was maximal at ISI = 40 ms in *Mkln1*^*–/–*^ slices (^**^P < 0.01) and decreased with larger intervals. *Mkln1*^*+/+*^ (*n* = 17 slices); *Mkln1*^*–/–*^ slices (*n* = 16 slices). **c**, **d** *Mkln1*^*–/–*^ mice showed impaired late-LTP induced by high-frequency stimulation (HFS) trains in the Schaffer collateral-CA1 region (arrows: 3× 1 s stimulations of 100 Hz at 0, 6, and 12 min). **c** The averaged fEPSP-slope values normalized to 30-min baseline. Despite comparable initial induction of LTP in *Mkln1*^*–/–*^ and *Mkln1*^*+/+*^ slices, the potentiation effect decayed more rapidly in *Mkln1*^*–/–*^ slices (time × genotype: *F*(90,1800) = 1.48, *P* < 0.01), leading an overall significant reduction in LTP (genotype: *F*(1,20) = 6.33, *P* < 0.05) by the end of a 4 h recording. Diamond points illustrate baseline recording in the CA1 region. Inset: Representative fEPSP traces (Dashed lines correspond to baseline while solid lines represent LTP). **d** Averaged fEPSP-slope values from 120–240 min (Late-LTP), which are significantly decreased in *Mkln1*^*–/–*^ slices (Independent *t*-test: t(20)= −3.18, *P* < 0.01). Data are represented as mean ± SEM. *Mkln1*^*+/+*^ (*N* = 7 mice); *Mkln1*^*–/–*^ (*N* = 8 mice). Baseline: *Mkln1*^*+/+*^ (*n* = 12 slices), *Mkln1*^*–/–*^ (*n* = 10 slices); HFS: *Mkln1*^*+/+*^ (*n* = 12 slices), *Mkln1*^*–/–*^ (*n* = 10 slices). **e** Immunoblots of fractionated extracts from hippocampal tissue showing separation into soluble (supernatant) and insoluble (pellet) fractions. Enrichment of synaptotagmin (Syt) and PSD-95 is shown in the soluble and insoluble fractions, respectively. **f**, **g** Analysis of actin levels in *Mkln1*^*+/+*^ and *Mkln1*^*–/–*^ hippocampal tissue lysates. **f** The upper panel shows representative blots of actin levels in the soluble and insoluble fractions. Protein levels were normalized to the housekeeping gene, Glyceraldehyde 3-phosphate dehydrogenase (GAPDH). **g** Compared with *Mkln1*^*+/+*^ tissue, actin protein levels were significantly increased in insoluble fractions of *Mkln1*^*–/–*^ hippocampal tissue (independent *t*-test with Welch’s correction: *t*(8.13) = 2.34, *P* < 0.05). *Mkln1*^*+/+*^ (*N* = 8)*; Mkln1*^*–/–*^ (*N* = 8) from 3 independent preparations. Data are expressed as a percentage of control ± SEM. **h** Representative images of *Mkln1*^*+/+*^ and *Mkln1*^*–/–*^ dissociated hippocampal neurons stained with phalloidin. Scale bar, 5 µm. **i** Frequency distribution showing F-actin immunoreactivity within phalloidin-stained regions of interest (ROI). The intensity of F-actin labeling in actin-rich compartments is significantly higher in *Mkln1*^*–/–*^ neurons than *Mkln1*^*+/+*^ controls (two-sample KS test: *D* = 0.24, *P* *<* 0.0001, *Mkln1*^*+/+*^: 1054; *Mkln1*^*–/–*^: 1067 dendritic protrusions). **j** Normalized phalloidin intensity measures were significantly increased in *Mkln1*^*–/–*^ actin-positive ROIs compared with *Mkln1*^*+/+*^ controls (independent *t*-test: *t* (38) = 2.88, *P* < 0.01). *Mkln1*^*+/+*^ (*n* = 20)*; Mkln1*^*–/–*^ (*n* = 20) neurons from two to three independent pre*p*arations. Data are expressed as a percentage of control ± SEM. **k**–**n** Elimination of muskelin leads to an increase in the spine F-actin stable pool. **k** FRAP analysis of GFP-actin 1 day after transfection of cultured hippocampal neurons (DIV 13); representative spine heads before and at different points after the laser bleaching impulse. Imaging and photobleaching conditions were similar for both conditions. Scale bar = 2 µm. **l** Analysis of GFP-actin fluorescence recovery shows that the plateau of the recovery curve in *Mkln1*^*–/–*^ spines does not approach the same level as *Mkln1*^*+/+*^ spines. **m** The stable actin fraction measured from FRAP curves of individual spines is significantly increased in *Mkln1*^*–/–*^ spines (genotype: *β* = 16.98, *t* = 5.49, *P* < 0.0001). **n** The GFP-actin recovery half-time of the dynamic F-actin pool is comparable for *Mkln1*^*–/–*^ and *Mkln1*^*+/+*^ spines. Scatter plots depict results per spine, and the line within bar graphs corresponds to the group mean value. *Mkln1*^*+/+*^ (*n* = 17 neurons, 47 spines), *Mkln1*^*–/–*^ (*n* = 25 neurons, 94 spines; 2 independent replications from four to six embryos per genotype.

Since a change in either mEPSC frequency or amplitude is sufficient to modify synaptic strength, we addressed whether muskelin deficiency might affect synaptic plasticity in the form of long-term potentiation (LTP) and long-term depression (LTD). Low-frequency stimulation (LFS) at the Schaffer-collateral pathway resulted in the comparable LTD in acute slices derived from both genotypes (Supplementary Fig. 4e). Similarly, the induction and expression of LTP following theta-bust stimulation (TBS) were indistinguishable between *Mkln1*^*–/–*^ and *Mkln1*^*+/+*^ slices (Supplementary Fig. 4f). Following high-frequency stimulation (HFS) patterns, the slope of the Input–Output (I/O) curve was similar in *Mkln1*^*–/–*^ and *Mkln1*^*+/+*^ slices (Fig. 6a). However, paired-pulse facilitation expressed as a function of interstimulus intervals was significantly higher in slices from *Mkln1*^*–/–*^ mice (Fig. 6b), suggesting that muskelin may be involved in synaptic potentiation mediated by presynaptic mechanisms. Despite a robust and equivalent induction of LTP by HFS in *Mkln1*^*–/–*^ and *Mkln1*^*+/+*^ slices, potentiation decayed more rapidly in *Mkln1*^*–/–*^ slices (Fig. 6c), leading to a significant impairment in synaptic potentiation in the late phase of LTP (Fig. 6d). Thus, it appears that muskelin contributes differentially to distinct forms of plasticity, with the loss of muskelin leading to enhanced short-term plasticity but deficits in late-phase maintenance of synaptic potentiation.

### *Mkln1* deletion leads to increased actin content and elicits changes in spine F-actin stability

The actin cytoskeleton and its dynamics are critical for establishing and maintaining dendritic arborization and spines, including spine structural changes during synaptic plasticity^9,34,35^. Since muskelin regulates cytoskeletal organization in adherent cell types^1,2^, we speculated that neuronal actin changes might be the candidate mechanism by which *Mkln1* deletion influences dendritic morphology, spine character, and synaptic functional properties. Therefore, we isolated hippocampal tissue and fractionated synaptosomal extracts to detect protein expression via western blotting. The enrichment of PSD-95 and synaptotagmin (Syt) confirmed the successful separation of insoluble (pellet) and soluble (supernatant) fractions, respectively (Fig. 6e). We found that the expression of PSD-95 and excitatory receptor subunits were unchanged in the insoluble fraction of *Mkln1*^*–/–*^ mice (Supplementary Fig. 4g, h). However, actin expression was significantly increased in the insoluble PSD-95 enriched fraction, with no differences detected in the soluble fraction (Fig. 6f, g). Analysis of the amount of immunofluorescent labeling in actin-rich compartments of primary hippocampal neurons delineated with phalloidin also revealed a significant increase in F-actin in *Mkln1*^*–/–*^ dendrites compared with controls (Fig. 6h–j).

Next, we employed fluorescence recovery after photobleaching (FRAP) in neurons transfected with GFP-tagged actin (13 *DIV*) to monitor actin filament dynamics and turnover in mature, well-defined dendritic spines. While selective photobleaching rapidly decreased GFP-actin fluorescence in single spines, we observed a marked reduction in the recovery of GFP-actin *Mkln1*^*–/–*^ spines (Fig. 6k, l). Compared with *Mkln1*^*+/+*^ spines, analysis of the recovery curves revealed a significant increase in the relative stable F-actin pool in *Mkln1*^*–/–*^ spines (Fig. 6m). Actin filaments in the dynamic pool recovered to the same extent in *Mkln1*^*–/–*^ and *Mkln1*^*+/+*^ spines (Fig. 6n), indicating that the predominant effect of *Mkln1* knockout on actin turnover is to increase the pool of stable actin in dendritic spines. Consistent with prior observations in non-neuronal cells^2^, these findings indicate that muskelin is required for actin regulation in neurons, which may be a critical contributing factor to the synaptic effects and functional outcome in *Mkln1*^*–/–*^ mice.

## Discussion

Given the limited knowledge about the relevance of muskelin in vivo, the present study identifies a critical role for muskelin in specific behavioral processes, including a significance in resting-state functional architecture and synaptic effects that underlie various cognitive and noncognitive functions. Behavioral phenotyping of *Mkln1*-null mice revealed an intact circadian locomotor profile consistent with the nocturnal nature of mice^36^. However, further assessment indicated that *Mkln1* knockout leads to maladaptive responses to novel settings or novel stimuli that trigger exploration. This is exemplified in the sustained locomotion despite repeated encounters to novel environments, suggesting an impaired ability to adapt to novelty or abnormalities in the motivation state of exploration. The latter is illustrated in decreased habituation of spontaneous exploratory behavior in a test that was free from confounding effects of forced exploration. Furthermore, *Mkln1* knockout alters exploratory drive as reflected in the heightened investigatory response elicited by a novel object. In rodents, novelty elicits exploratory behavior, representing a mode to gather information about the environment to guide future adaptive behavior^14^. The subsequent decline in exploratory activity over time as the context loses its novelty (habituation) is considered a form of non-associative learning^14,37^. While the intense exploration in *Mkln1*^*–/–*^ mice may reflect an  increased pursuit of novel environmental features, the lack of habituation suggests decreased capacity to acquire sufficient information that may impair learning or recall about the environment or novel stimuli.

Functional neuroimaging indicated that the underlying neural mechanism responsible for some of the behavioral outcomes in *Mkln1*^*–/–*^ mice might lie in aberrant resting-state functional connectivity. We did not observe significant genotype differences in DTI-MRI measures, suggesting that in *Mkln1*^*–/–*^ mice, water diffusion changes are mild despite altered bilateral and homotopic functional coupling. In particular, *Mkln1* knockout reshapes the connectional profile of the hippocampus, resulting in weaker or more robust functional connectivity with regions implicated in locomotor exploration, social cognition, and fear regulation. Muskelin is highly expressed in the hippocampus^5^, where it is shown to influence hippocampal neural activity patterns^7^, thus supporting the prediction for the hippocampus as a critical substrate for specific behavioral changes in *Mkln1*-null mice. Here, deficient hippocampal-striatal functional connectivity may account for the phenotypes in the open-field assays (Fig. 1). When placed in a novel environment, rodents form a hippocampal-dependent internal representation of the surrounding spatial information^37^. Environmental context representations require striatal involvement, which acts in conjunction with the hippocampus to encode spatial context and movement information in familiar environments^38^. Furthermore, the striatum’s capacity to encode both locomotion and environmental identity indicates that hippocampal-dependent information regarding context identity influences the animal’s decision to explore the environment via the dorsal striatum^39^. Functional disconnection between the hippocampus and dorsal striatum in *Mkln1*^*–/–*^ mice may impair exploratory learning, resulting in the subsequent recovery of locomotor exploration upon re-exposure to a novel environment.

We found that while *Mkln1*^*–/–*^ mice could maintain a preference for social over inanimate objects, they show indiscriminate interaction with familiar and novel social stimuli. Considering our observations in the open-field assays, the social memory impairment may stem from impaired habituation of exploratory behavior. The renewed interest directed towards the familiar conspecific (in the recall phase) suggests that inadequate habituation to a previously encountered social stimulus may compromise the biased preference for novel social stimuli in subsequent encounters. Alternatively, the observation might reflect deficits in novelty acquisition, in which a failure to gather sufficient information related to a social stimulus in a prior encounter interferes with the ability to recognize social novelty afterward. The presence of this phenotype strongly correlates with aberrant functional connectivity involving hippocampus/mPFC/ACC/amygdala circuits in *Mkln1*^*–/–*^ mice. Social recognition memory is encoded by an interregional functional network composed of these four regions, with the hippocampus playing a crucial role in coordinating regional interactions and generating social recognition memory^28^. Notably, the present pattern of behavioral outcomes coincides with impaired high-frequency-induced hippocampal long-term potentiation (HFS-LTP) in *Mkln1*^*–/–*^ mice, in keeping with previous studies demonstrating a pivotal role for HFS-LTP in novelty acquisition and exploration of novel environments^40,41^.

It has been demonstrated that dendritic spines undergo morphological changes during synaptic plasticity, with the actin cytoskeleton and its dynamic properties playing a critical role in spine structural changes^9,34,35^. Here, we identify muskelin localization in asymmetric (excitatory) synapses and demonstrate a novel role for muskelin in regulating the actin stable pool in dendritic spines. Importantly, we show that spines depleted of muskelin have enlarged spines heads but are also significantly shorter than control spines. Previous work has demonstrated that LTP can increase the size of dendritic spine heads while inducing a simultaneous decrease in spine length^42^. Furthermore, evidence indicates that LTP induces a shift towards more stable actin filaments, leading to larger spine volumes and increased postsynaptic binding capacity^43^. One possibility is that under basal conditions, the impact of *Mkln1* knockout on spine head size and length already mimics the effects induced by LTP, impeding further spine structural changes necessary for post-tetanic potentiation. Based on prior indications of its role in regulating myosin II activity^2^, it is plausible that muskelin participates in neuronal F-actin dynamics via actomyosin interactions. We propose that loss of muskelin may disrupt myosin II remodeling activity on spine actin, leading to more stable F-actin filaments and corresponding changes in dendritic spine morphology. Besides actin’s structural role in spine morphology, dynamic F-actin is critical for AMPAR trafficking during synaptic plasticity^44^. The loss of muskelin and consequent increase in stable actin may facilitate synaptic function by increasing AMPAR anchoring at the post-synapse under basal conditions but impair activity-dependent insertion of new AMPARs at the membrane. The prominent HFS-induced late-LTP deficits seen in *Mkln1*^*–/–*^ slices might therefore reflect convergent effects of hyper-stable spine actin and decreased dynamic exchange of AMPARs at synapses. Each outcome could negatively impact synaptic potentiation and interfere with mnemonic functions that rely on exploration-habituation, including memory formation of novel experiences in which HFS-induced LTP is implicated^40,41^.

While synaptic plasticity is considered a cellular substrate for certain forms of learning and memory^45^, it is unlikely that hippocampal long-term synaptic potentiation is the primary mechanism responsible for memory capabilities beyond environmental novelty exploration and social recognition in *Mkln1*^*–/–*^ mice. Indeed, increasing evidence has challenged a causal relationship between LTP and specific memories; some studies report a positive correlation while others have failed to identify a significant role for LTP in spatial learning^46^. One explanation leading to enhanced consolidation of spatial and fear-extinction memories in *Mkln1*^*–/–*^ mice may lie on the impact of *Mkln1* knockout on synchronized neural network activity. Field recordings in hippocampal slices derived from *Mkln1*-null mice have revealed a profound increase in the amplitudes of spontaneously occurring sharp-wave ripples (SPW-Rs)^7^. Hippocampal SPW-Rs are reactivated during quiet wakefulness or slow-wave sleep^47^ and shown to underlie memory consolidation of spatial memories^48,49^ and fear-extinction memories^50^. Based on our current cellular findings, it is likely that the enhancing effect of *Mkln1* knockout on synchronized network activity emerges from the interplay between modifications in neuronal structural and functional properties. One prediction for increased SPW-R amplitudes upon *Mkln1* knockout is reduced inhibition of target principal neurons due to more tonic inhibition generated by increased extrasynaptic GABA_A_ receptor levels on basket cells^7^. Second, the marked increase in AMPAR-mediated mEPSCs in *Mkln1*^*–/–*^ neurons may augment neuronal firing synchrony since larger SPW-R amplitudes are associated with increased synchronous excitation (excitatory postsynaptic potentials, EPSPs) in CA1 pyramidal neurons^51,52^. Enhanced network activity could arise from increased synaptic facilitation (Fig. 6b), which is reported to influence the dynamics of synchronized hippocampal network activity^53^. Finally, an increase in ripple amplitude reflects the recruitment of a larger population of hippocampal neurons in an SPW-R-associated synchronous discharge^54,55^. Since synchronized network activity relies on neural connectivity, the increased dendritic branch arborization in *Mkln1*^*–/–*^ neurons may enhance neuronal interconnectivity and promote synchronized activity in *Mkln1* knockout neural ensembles. The cumulative changes involving neuronal morphology and AMPAR-mediated synaptic excitability may contribute to increased network activity in *Mkln1*^*–/–*^ mice and correlate with superior functioning in cognitive domains such as spatial learning and fear-extinction recall. At the same time, our collective data suggest that the enhancing effect of *Mkln1* knockout on local network activity may occur at the expense of more distant connections. We speculate that one consequence of anomalous hippocampal activation is a general readjustment of brain functional coupling dynamics; such changes point to an adaptive compensatory response to limit overexcitability resulting from hyperconnected local neural networks.

While this study focused on the relevance of muskelin in behavioral functions and identifying neural and synaptic correlates, we anticipate that *Mkln1*-null mice may be instrumental in understanding the biological basis of disorders in which impaired exploratory activity and cognition are key features. A previous genome-wide association study (GWAS) showed that the human muskelin gene (*MKLN1*) variant (rs114034759-A) is significantly associated with increased risk of early-onset bipolar disorder and lower expression of MKLN1 protein levels in the hippocampal brain region^56^. It was suggested that lower hippocampal MKLN1 expression and the corresponding increase in hippocampal excitability might lead to an increased risk of developing a manic/hypomanic episode^56^. In particular, the novelty-induced hyperlocomotion and exploratory profile of *Mkln1*^*–/–*^ mice (Fig. 1) resembles the specific and goal-directed exploration characteristic of the manic state. Previous investigations using the human version of the rodent open field (human behavioral pattern monitor) have identified distinct characteristics of schizophrenic (SCZ) and manic BD patients based on activity, exploration, and interaction with objects^57^. When exposed to a novel exploratory environment, manic BD patients show increased locomotor activity compared with SCZ patients^58,59^, but SCZ patients fail to show habituation of motor activity across time^59^. Furthermore, manic BD patients exhibit increased exploration characterized by greater physical interactions with novel objects and more object perseveration than SCZ patients^59,60^.

Although we cannot entirely exclude a potential relevance to other hyperactivity-related disorders that share a broad overlap in behavioral features^61,62^, specific endophenotypes and pharmacological responses can be employed to distinguish an animal model for one disorder over another^20^. Indeed, some of the phenotypes reported here are inconsistent with recognized behavioral correlates of psychosis and ADHD rodent models^63–65^. For instance, we demonstrate diminished response to the motor stimulating effect of amphetamine, which attenuates hyperactivity associated with ADHD in patients^66^ and animal models^65^. Enhanced response to amphetamine is reported in schizophrenic patients^67^ and is considered an endophenotype related to the positive symptoms of SCZ in animal models^63^. Moreover, *Mkln1*-null mice showed normal prepulse inhibition (PPI) and startle habituation, features that are impaired in schizophrenic patients^68^ and manic patients with psychosis^69^.

While stable euthymic BD patients are reported to have enhanced fear-extinction memory^70^, we also note that some of the phenotypes in *Mkln1*-null mice do not mirror notable features of mania or BD. Cognitive deficits, especially those associated with vigilance and emotional regulation, are commonly associated with mania^71,72^, and a lesser extent, verbal and spatial working memory^73^. The intact circadian locomotor profile in *Mkln1*^*–/–*^ mice is inconsistent with altered circadian rhythms and sleep disturbances in patients with BD^74^. Given the complexity of BD and the high number of candidate genes implicated in the disorder^75–77^, no single gene defect can be entirely attributed to its manifestation. Hence, the *Mkln1* gene, which acts through specific neuronal and synaptic mechanisms, is likely to exert its effects on distinct brain functions as well as specific phenotype dimensions or behavioral features of BD.

In summary, this study demonstrates the critical role of muskelin in exploratory activity and memory formation, including a relevance in the functional connectivity of resting-state brain networks that underlie these processes. Muskelin is localized within dendritic protrusions and asymmetric excitatory synapses, where it plays an essential role in AMPAR-mediated synaptic transmission and the capacity of CA1 synapses to maintain synaptic potentiation. We highlight a role for muskelin in regulating actin stability in dendritic protrusions and show that muskelin is necessary for neuronal morphological properties related to dendritic ramification and spine structural characteristics. Altogether, our data show that in vivo phenotypes arising from muskelin loss-of-function may be centered upon aberrant regulation of actin stability and corresponding synaptic effects.

## Methods

### Mice

The generation of muskelin homozygous knockout (*Mkln1*^–/–^) mice is described elsewhere (Heisler et al.^7^). The *Mkln1*^–/–^ mouse line was backcrossed seven generations into the C57BL/6 background before testing. Mice were housed in groups of 3–5 mice per cage in a temperature-controlled (20–22 °C) vivarium and maintained under an inverted 12-h light/dark cycle (lights on 7:00 pm–7:00 am). Food and water were available *ad libitum*. Behavioral experiments were conducted during the dark (active) phase of the cycle. All animal studies complied with the European Communities Council Directive (2010/63/EU) on the protection of experimental animals and guidelines set forth by the German Animal Welfare Act. Experiments were conducted following approval by the ethics committee of the City of Hamburg (Behörde für Gesundheit und Verbraucherschutz, Fachbereich Veterinärwesen; No. 68/15 and No. 106/10). Behavioral experiments were conducted on male and female mice. The number of mice used per genotype and sex is provided in the corresponding figure legend for each experiment.

### Elevated plus maze

The apparatus and testing in the elevated plus maze have been described previously^78^. For each trial, the mouse was placed in the central square facing one of the open arms and allowed to explore the maze for 5 min. The tracking and analysis of behavior during trials were carried out using the Ethovision computerized tracking software (Version XT 8.5, Noldus Technology, The Netherlands). The proportion of time spent in the open arms was used as an index of anxiety-related behavior and calculated according to the following formula: %Time = (time in open arms/(time spent in open arms + time spent in closed arms)) × 100).

### Light–dark transition test

This test was used as an additional measure of anxiety-related behavior and capitalized on rodents’ natural preference for dark enclosed areas over more aversive open and brightly lit areas^79^. The apparatus consisted of a rectangular white box (45 × 20 × 20 cm; length × width × height) made of PVC foam board. It was partitioned into two compartments consisting of a dark chamber (15 × 20 × 20 cm) and an “open” area (30 × 20 × 20 cm). A 7.5 × 7.5 cm opening was positioned in the middle of the partitioning wall to facilitate the transition between the lit and dark compartments. The lit chamber was brightly illuminated (390 lux), whereas the dark enclosure was dimly illuminated (1 lux). The mouse was placed in the middle of the brightly lit compartment and freely explored the arena for 10 min. Automated tracking was carried out using Ethovision. The time spent in the brightly lit compartment was taken as a measure of anxiety-like behavior.

### Locomotor activity and habituation in the open field

Testing was conducted in four identical arenas, as previously described^80^. Mice were tested in four consecutive 30-min sessions separated by 24-h intervals to examine locomotor activity and habituation. Tracking and trial analysis were conducted using Ethovision video tracking software. The arenas were cleansed with 70% EtOH between trials. Locomotor activity was indexed as distance traveled in 5-min consecutive bins. In addition, time spent in the arena’s central zone was calculated as an index of anxiety-related behavior. To evaluate locomotor habituation within each test session, an activity change ratio (distance traveled in the last five min/ (distance in the first 5 min + distance in the last 5 min)) was calculated as defined previously^14^. Between-session habituation was calculated as follows: distance moved on day *n*/(distance moved on day 1 + distance moved on day *n*).

### Free choice exploration in the open field

Testing was carried out in two identical arenas (50 × 50 × 50 cm high) made of waterproof PVC foam board material. A square opening (6 × 6 cm) positioned in the middle of one arena wall allowed free access to a dark escape compartment (16 × 16 × 16 cm high). The arenas were illuminated by lamps that provided diffuse (50 lux) even lighting in each arena. Test sessions were recorded using the Ethovision tracking software. For two days before testing, access to the main arena was blocked, and the mouse habituated to the dark compartment for 5 min each day. Mice were then introduced to the dark compartment with free access to the main arena and tested in two consecutive 1-h sessions separated by a 24-h interval. Arenas were cleansed with 70% EtOH between trials. The total distance moved relative to time spent in the open arena was taken as an index for locomotor activity.

### Novel object-elicited investigation in the open field

Testing was conducted in open-field arenas as described previously^80^. Mice were introduced to the arena and allowed to explore undisturbed for 30-min. They were briefly removed from the open fields, during which four identical objects were fixed in the center of each arena. Mice were re-introduced to the arena and tested for an additional 30 min. All sessions were recorded using the Ethovision video tracking software (Version XT 8.5, Noldus Technology, The Netherlands) equipped with three-point (nose-point, center-point, and tail-base) detection settings. Arenas and objects were cleaned with 70% EtOH between trials. As an index of active object exploration, the time spent in contact with the object was scored when the mouse´s nose was located within a 2-cm radius around the object. Latency to approach was indexed as the time elapsed between introducing the object and first contact with the object. Locomotor activity was indexed as the distance moved as a function of 5-min consecutive bins.

### Circadian locomotor activity in the home cage

Testing was conducted in Type II polycarbonate cages (20 × 26 cm), each equipped with a Mouse-E-Motion unit (Mouse-E-Motion; Infra-E-Motion GmbH, Hamburg Germany). The device was configured to detect motion using an infrared sensor with a sampling frequency of 1 s and a 4-min recording interval of cumulative activity. Data were transmitted to a PC through the Mouse-E-Motion Universal Data Logger with an Excel add-in that provided the functionality to download collected log data. Mice were individually housed and allowed to habituate to the new cages one week before testing. Activity levels were monitored across the 12-h light/dark cycle for three consecutive days. Lights ON is denoted by zeitgeber (ZT) 12 and lights OFF by ZT0, with the transition between the light and dark phases occurring progressively during a 1 h period. To estimate the circadian rhythm, a model consisting of cosine curves with 24-h periods was fitted by least squares to the activity data (1-h bins) using the Cosinor program^81^. An estimate of the central tendency of activity distribution (Midline Estimating Statistic of Rhythm (MESOR) was calculated.

### Test for sociability and social novelty preference

The 3-chamber social paradigm was employed to examine general sociability and cognition in the form of social novelty preference^19^. The apparatus and detailed protocol are described previously^82^. A discrimination index used to assess social memory was calculated according to the following formula: Discrimination index = [(Time_Novel_ – Time_Familiar_)/Time_Novel+Familiar_]. The calculation leads to a score ranging from −1 (preference for the familiar conspecific) to +1 (preference for a novel mouse). One-sample *t*-tests were used to compare the discrimination measure of each genotype group with zero (random exploration value). An additional analysis was conducted to compare the level of discrimination between genotype groups.

### Locomotor response to amphetamine

Amphetamine (Amph) sulfate (Sigma-Aldrich) was dissolved in 0.9% NaCl solution on the day of testing to achieve 2.5 mg/kg in a volume of 5 ml/Kg. This dose was chosen to induce a measurable locomotor response in mice, with minimal stereotypic or ataxic behaviors^83^. The experiment was carried out in the open-field apparatus, as described above. Basal locomotor activity was recorded for 30 min before intraperitoneal (i.p.) administration of either saline (0.9% NaCl) or Amph (2.5. mg/kg). Mice were afterward observed for 2 h to assess locomotor response to Amph. Post-injection data were normalized to baseline locomotion to minimize potential confound from differences in basal locomotor activity.

### Testing for the acoustic startle reflex

#### Apparatus

Testing was conducted in two Startle Reflex Lab (SR-LAB) systems (San Diego Instruments, San Diego, CA, USA), each comprising a sound-attenuating chamber, a light source, and a sound generator that emitted continuous background noise (65 dB) and white-noise pulses of different sound intensities. Each chamber contained a test platform consisting of a clear cylindrical mouse enclosure mounted on a Plexiglas base connected to a piezoelectric accelerometer sensor. Startle calibration and chamber standardization were conducted before testing by mounting an SR-LAB Standardization Unit (San Diego Instruments) onto the animal enclosures to attain a reasonable waveform response (700 ± 15 Millivolts) and to ensure that a given baseline response was similar for both test chambers. Whole-body responses were transduced by the piezoelectric accelerometer sensor, digitized, rectified, and transmitted to a computer running the SR-LAB software. Startle reaction was analyzed by measuring the maximum (Vmax) of the waveform response (from 0 to 65 ms) to every trial stimulus.

#### Acoustic startle response

Startle reactivity was examined in a session that lasted ~30 min. Mice were introduced to the test chambers and first acclimatized to background white noise (65 dB) for 5 min. Thereafter, mice were randomly presented with white-noise pulses of varying intensity. Trials included background noise alone (65 dB) and 40-ms acoustic stimuli (69, 73, 77, 81, 85, 90, 95, 100, 110, 120 dB) presented with a variable intertrial interval ranging from 10 to 20 s (average 15 s). The maximum response for each stimulus (Vmax) was plotted against sound intensity.

#### Prepulse inhibition (PPI)

Mice were examined for PPI using a three-by-three (3 pulse stimulus, 3 prepulse intensities) experimental design, as recommended by Yee et al.^23^. This approach is shown to cater to baseline startle differences between groups, thus allowing a critical assessment of prepulse inhibition (PPI) in the event of confounding differences in startle reactivity^84^. Mice were first acclimatized to the test chambers for 5 min and then presented with six pulse-alone trials to habituate and stabilize the startle response. This was followed by presenting ten main blocks of trials, each comprising a mixture of sixteen discrete trial types: three pulse-alone trials, three prepulse-alone trials, nine prepulse-plus-pulse trials, and one trial in which no discrete stimulus other than the constant background noise was presented. Discrete trials within each block were presented in a pseudorandom order with a variable intertrial interval ranging from 10 to 20 s. Acoustic stimuli were in the form of white noise with a rise time of ~1 ms presented against a constant background noise level of 65 dB. The three pulse stimuli were 40 ms in duration and 100, 110, or 120 dB in intensity. Prepulse stimuli (71, 77, or 83 dB) were 20 ms in duration, corresponding to +6, +12, or +18 dB above background noise. There were, therefore, nine possible combinations of prepulse-plus-pulse trials, for which a stimulus onset asynchrony (SOA) of 100 ms between the two stimuli was used. The test session concluded with the presentation of six pulse-alone trials and lasted approximately 48 min. The first and final three startle amplitude blocks were analyzed separately to assess the initial and final habituation of startle responses. Percentage prepulse inhibition (PPI) for each prepulse trial (71, 77, or 83 dB) was calculated according to the following formula: %PPI = [(startle amplitude_pulse_ – startle amplitude_prepulse+pulse_)/startle amplitude_pulse_) × 100].

#### Habituation of the acoustic startle response

Mice were introduced to the chambers for a 5-min acclimatization period with 65 dB of background noise. Fifty startle stimuli (120 dB, 40 ms) were presented with a variable intertrial interval ranging from 10 to 20 s (average 15 s). The maximum response to the stimulus (Vmax), averaged for blocks of 5 stimulus trials, was analyzed to determine startle response habituation.

### Morris water maze spatial reference memory

The Morris water maze apparatus and procedure involving acclimation to water are described in detail elsewhere^78,80^.

#### Non-spatial cued platform test

Here, the pool was surrounded by curtains to occlude extra-maze cues and the submerged platform indicated by a prominent cue. This procedure served to control potential sensorimotor deficits and the motivation to swim. Subjects were trained for one day across four consecutive trials (ITI = 8 min) to navigate to the visible platform. Mice were released from a constant location (S), but the location of the visible platform was varied in a pseudorandom manner across trials. The platform was positioned in the middle of the three quadrants and the pool’s center, but the quadrant designated from hidden platform training was not used (Cued platform: SW, C, NE, SE; target hidden platform: NW). A trial ended when mice successfully climbed onto the platform or after 60 s had elapsed, at which point they were guided to the platform by the experimenter. Mice were allowed to remain on the platform for 15 s before being taken out and briefly dried with a towel. Mice were housed in waiting cages placed on a heating pad between trials.

#### Hidden platform test

Mice were then trained to utilize extra-maze cues to navigate- and escape to a platform submerged 0.5 cm below the water surface. The hidden platform was constantly located in the middle of the target training quadrant throughout training. Mice received four trials per day (ITI = 10 min) across seven days of training (Acquisition). The release points for each trial were varied in a pseudorandom sequence. A trial ended when the mouse successfully climbed onto the platform or after 60 s had elapsed, during which the experimenter gently guided it to the platform. Mice were allowed to remain on the hidden platform for 15 s before being removed from the pool.

#### Probe test

Twenty-four hours after training, spatial memory for the new training quadrant was examined in a 90-s probe test with the hidden escape platform was absent from the pool. At the end of the trial, mice were removed from the pool and allowed to completely dry in waiting cages placed on a warm heating pad. Search preference in the training quadrant was considered an index of successful place memory acquisition.

#### Variables analyzed

Due to significant differences in swim speed between the two genotype groups, spatial learning was inferred by computing swim track distance (path length) as the main dependent variable as it is independent of any confounds in swim speed. The criterion adopted for the search strategy analysis during the probe trial included the proportion of swim track (% path length) within the target quadrant. We also computed the subject’s proximity to the escape platform location throughout the search (mean search error). The proximity measure provides a more reliable index of search accuracy and spatial bias^26^.

### Auditory cued fear conditioning and fear extinction

The testing apparatus comprised two identical NIR video fear conditioning system (MED-VFC-SCT-M, Med associates Inc, VT, USA). The training chambers (30 × 24 × 21 cm) were composed of aluminum sidewalls, a clear Plexiglas front door, and were housed in sound-attenuating cabinets. Each chamber was equipped with a stainless-steel grid floor (19 stainless-steel rods, 4 mm in diameter and spaced 1.5 cm apart) through which an electric shock (unconditioned stimulus, US) could be delivered. A 15 W house light mounted above the grid floor on one side wall illuminated the testing chamber. On the opposite wall was a speaker that was used to deliver acoustic stimuli (e.g., a pure tone, conditioned stimulus). Video images during testing sessions were recorded (30 frames/s) and transmitted to a computer running the Video Freeze software (Med Associates). The house light was turned on during conditioned acquisition, and a distinct vanilla odor was used to provide distinct olfactory cues (Context A). The training chambers were modified during extinction training by adding a black triangular acrylic insert and a smooth white acrylic insert instead of the grid floor (Context B). To further maximize discrimination between the two contexts, the house light was turned off during testing sessions, and trials were recorded under Near Infrared (NIR) light. The chambers were cleaned with 70% EtOH between trials. We measured freezing behavior as the conditioned response (CR), which is defined as the cessation of all movement apart from respiration.

#### Conditioned acquisition and extinction retention

On the first day of training, mice were introduced to the conditioning chambers (Context A) and allowed a 3 min exploration interval to establish baseline freezing levels. Thereafter, the mice were given three conditioned CS-US trials interspaced by 3-min intertrial intervals. Each trial comprised the presentation of a 30-s discrete tone (Conditioned stimulus (CS); 90 dB, 2.8 kHz). Upon immediate termination of the tone, a 1-s 0.4 mA foot shock (unconditioned stimulus (US)) was delivered through the grid floor. This was followed by a 3 min stimulus-free interval before the mice were removed from the chambers and returned to their home cages. On the following day, mice were examined in a modified and neutral context (Context B). Following a 2-min context acclimation period, mice received 10 × 30-s CS presentations interspaced by 20-s intertrial intervals. Repeated testing across five days was used to examine both within-session and between-days extinction to non-reinforced CS trials. Remote extinction memory retention was tested in context B, 21 days after the last extinction training day. Mice received 10 × 30-s CS presentations interspaced by 20-s intertrial intervals. Context fear renewal was tested by re-introducing mice to the original training context (context A). Following a 3-min acclimation period, mice received 3 × CS-only presentations interspaced by 3-min intertrial intervals.

### Magnetic resonance imaging (MRI)

#### Acquisition

Experiments were performed on 19 male mice (*Mkln1*^*+/+*^ (*N* = 8)*; Mkln1*^*–/–*^(*N* = 11) at 22–24 weeks of age. Animals were scanned using a 7T small animal MRI (Clinscan, Bruker, Ettlingen, Germany) consisting of a ^1^H receive-only 2 × 2 mouse brain surface array coil and a ^1^H transmit volume coil (inner diameter of 72 mm). Animals were under mild anesthesia during scanning (~1% isoflurane (Baxter, Munich, Germany) in oxygen (0.5 l/min)). Body temperature was maintained by a heating pad circulating 37 °C warm water. The respiration rate was monitored by a small animal vital sign monitor (SA Instruments Inc., Stony Brook, NY), and the isoflurane concentration was adjusted around 1% to maintain a respiration rate of 100/min for all animals during the scan. Mice were placed in a dedicated MRI holder, which immobilized the head and allowed a close fit of the receive coil to the animal’s head. Resting-state functional MRI (rsfMRI) was acquired by single-shot echo-planar imaging (EPI) with the following parameters: echo time (TE) = 9 ms, repetition time (TR) = 2.5 s, 150 repetitions, voxel size = 312 × 312 µm, image matrix = 64 × 64 with 6/8 partial Fourier acquisition factor in the phase encoding direction, frequency encoding bandwidth = 3125 Hz/pixel, 24 slices, slice thickness 300 µm, 100 µm gap between slices, total acquisition time 6:20 min. Diffusion tensor MRI (DTI) was acquired by single-shot spin-echo (SE) EPI with the following parameters: diffusion weighing value b = 1000 s/mm^2^ in 12 non-collinear directions and two averages and two additional b = 0 acquisitions, echo time (TE) = 43 ms, repetition time (TR) = 15 s, voxel size = 156 × 156 µm, image matrix = 128 × 128 with 5/8 partial Fourier acquisition factor in the phase encoding direction, frequency encoding bandwidth = 2790 Hz/pixel, 24 slices, slice thickness 400 µm, total acquisition time 6:45 min.

#### Analysis

Mean diffusion (MD) and fractional anisotropy (FA) maps were reconstructed using DTIFIT of the FMRIB software library (FSL). Anatomical b = 0 images were realigned to a mean b = 0 image of all animals using the FSL image registration tools FLIRT^85,86^ and FNIRT. The image registration was repeated twice with an updated mean b = 0 image of all previously realigned images to improve the registration results. The rsfMRI images were also registered using FLIRT and FNIRT to the final mean b = 0 image. Regions of interest were defined in the anatomical mean b = 0 image and applied to the realigned MD, FA, and rsfMRI images. The MD and FA maps were compared voxel-wise between the two animal groups using a two-sample t-test in Matlab (Natick, MA, USA). Matlab was also used for the rsfMRI analysis. The mean ROI signal of the realigned rsfMRI data was corrected for linear drift and high-pass filtered (cut-off frequency at 0.01 Hz). The correlation of the signal between all ROIs was analyzed for each group by partial correlation controlling for the non-functional signal fluctuations in the ventricles and WM ROIs. This was done to minimize a false positive correlation due to overall signal effects caused by respiration or pulsation. The Fisher r-to-z transformation was used to compare the ROI correlations between the two groups.

### Acute hippocampal slice preparation and electrophysiological recordings

Brains from *Mkln1*^*–/–*^ and *Mkln1*^+/+^ mice were dissected using a vibratome (LeicaVT1000S, Nussloch, Germany) into 350-μm-thick slices. The acute hippocampal slices were incubated at room temperature for 2 h in carbogenated (CO_2_ 5%, O_2_ 95%) artificial cerebrospinal fluid (ACSF, containing in mM: 110 NaCl, 2.5 KCl, 2.5 CaCl_2_·2H_2_O, 1.5 MgSO_4_·7H_2_O, 1.24 KH_2_PO_4_, 10 glucose, 27.4 NaHCO_3_, pH 7.3). The slices were then transferred into a recording chamber (at 31 ± 1 °C). Field excitatory postsynaptic potentials (fEPSPs) were evoked by stimulation of CA1 Schaffer-collateral fibers with stainless electrodes. fESPSs were recorded with ACSF-filled glass capillary microelectrodes (3–5 MΩ), amplified by an Extracellular Amplifier (EXT-02B, NPI electronic, Germany), and digitized at a sampling frequency of 5 kHz by Digidata 1401plus AD/DA converter (CED, England). Data were acquired with the PWIN software (LIN, Magdeburg, Germany). The width of the single stimulus was 0.2 ms. The Input–Output (I/O) curve was tested with different stimulus intensities, and the paired-pulse facilitation (PPF) was tested with different interstimulus intervals. Afterward, single stimuli were applied every 30 s (at 0.0333 Hz) during long-term recordings and were averaged every 3 min. Stimulation strength was adjusted to 30–40% of the maximum fEPSP-slope values for the long-term potentiation (LTP) experiments. After 30 min of stable baseline recording, LTP was induced by tetanization with: (a) high-frequency stimulations (HFS, consisting of three 1 s stimulus trains at 100 Hz with a 6 min inter-train interval), or (b) theta-burst stimulations (TBS, consisting of 5 trains theta burst with 200 ms inter-train interval and each burst contains four pulses at 100 Hz). Stimulation strength was adjusted to 40%~50% of the maximum fEPSP-slope values for the long-term depression (LTD) experiments. After 30 min of stable baseline recording, LTD was induced by low-frequency stimulations (LFS, consisting of 900 pulses at 1 Hz).

### Whole-cell patch-clamp recordings

Hippocampi were dissected from 16-day *Mkln1*^*–/–*^ and *Mkln1*^+/+^ embryos (E16) and primary neuronal cultures prepared as previously described^80,87^. Whole-cell patch-clamp measurements^88^ were performed on DIV 20–21 neurons. Recordings were made using borosilicate pipettes with resistances of 3.0–4.5 MΩ after filling with intracellular solution (120 mM K-gluconate, 8 mM NaCl, 2 mM MgCl_2_, 0.5 mM CaCl_2_, 5 mM EGTA, 10 mM HEPES, 14 mM phosphocreatine, 2 mM magnesium-ATP, 0.3 mM sodium-GTP, and pH adjusted to 7.3 with KOH). The patchmaster software (HEKA, Lambrecht, Germany), in combination with an EPC-9 patch-clamp amplifier (HEKA), was used for data acquisition and pulse application. Recordings were low-pass filtered at 2.9 kHz and analyzed with Fitmaster (HEKA), Igor Pro 6.03 (Wavemetrics), Mini Analysis (Synaptosoft, Decatur, GA), and Excel (Microsoft). Neurons with an access resistance of <20 MΩ were evaluated. Miniature excitatory postsynaptic currents (mEPSCs) were recorded for 30 s and processed with the Mini Analysis software (Synaptosoft). Experiments were done at room temperature (21–23 °C) in Ringer´s solution (143 mM NaCl, 5 mM 1 KCl, 0.8 mM MgCl_2_, 1 mM CaCl_2_, 10 mM HEPES, 5 mM glucose, and pH adjusted to 7.3 with NaOH). mEPSCs were recorded in the presence of TTX (0.25 μM), bicuculline (10 μM), and AP5 (20 µM) added to the control solution. Current clamp recordings were performed in Ringer´s solution in the absence of the drugs. All substances were purchased from Sigma-Aldrich.

### DiOlistic (DIL) dye labeling in fixed brain slices for analysis of neuronal morphology

Mice from both genotypes were perfused with 4% PFA and 0.1% glutaraldehyde in PBS. Hippocampi were sectioned into 300-µm slices, which were maintained in fixative until ready for use. The procedures involving the DiOlistic labeling of neurons and image acquisition are described in detail previously^80^.

#### Dendritic tree analysis

Quantifications were carried out from non-overlapping CA1 pyramidal neurons. Dendritic arbors from confocal image stacks (2-µm step sizes) were manually traced using the Simple neurite tracer plugin^89^ in FIJI (Image J, NIH). Sholl analysis^90^ was carried out using the 3DSholl analysis plugin in FIJI, where a series of concentric circles were defined automatically from the edge of the soma at 10 µm of intervals. The maximum value of sampled intersections reflecting the highest number of branches in the arbor was calculated, and the number of intersections was plotted against the distance from the some in µm.

#### Dendritic spine analysis

Prior to analyses, high-magnification confocal image stacks (1-µm step size) of basal and apical dendritic segments were blindly deconvolved in AutoQuant Deconvolution software (X3, MediaCybernetics). The deconvoluted confocal images were used for the analysis of spine morphology and density in 3D using the NeuronStudio software^91^. Dendritic shafts were reconstructed automatically. The automated recognition and classification of spines were also confirmed manually. The number of spines per dendrite length and parameters related to spine morphology (spine length and spine head diameter) were automatically calculated in NeuronStudio.

### Immunocytochemistry and analysis of neuronal morphology

Immunofluorescence staining was carried out on cultured hippocampal neurons (DIV 21) derived from *Mkln1*^*–/–*^ and *Mkln1*^+/+^ mice. The cultures were rinsed with ice-cold phosphate-buffered saline (PBS) and fixed in 4% paraformaldehyde (PFA, w/v) and 4% sucrose (w/v) for 10 min. The cells were then washed three times with PBS, permeabilized with 0.25% Triton X-100 for 4 min, and rinsed further with PBS. Cells were incubated in blocking buffer (PBS containing 1% BSA) followed by incubation with primary antibodies (diluted in blocking buffer) for one hour at room temperature. Primary antibodies included mouse anti-MAP2 (1:500; SYSY, Goettingen, Germany). Coverslips were washed three times with PBS and incubated for 1 h at room temperature with goat anti-mouse Alexa 488 (1:500, Dianova, Hamburg, Germany) antibody. After three rinses in PBS, neurons were counterstained with Phalloidin Rhodamine (1:500; TebuBio, Offenbach, Germany) and DAPI (1:1000). Coverslips were rinsed one final time with PBS and mounted on slides using Aqua poly mount (Polysciences, PA, USA).

#### Image acquisition

Images were acquired using an Olympus FlouView TMFV1000 laser scanning confocal microscope (Olympus, Hamburg, Germany) equipped with ×20 (NA 0.85) and ×60 (NA 1.35) oil immersion objectives. The microscope was equipped with argon 488 nm, DPSS 561 nm, and He-Ne 594 nm lasers and connected to a computer running the FluoView (Version 4.2) software. For double- or triple-channel fluorescence, images were acquired separately using sequential scanning mode under identical exposure conditions. The z-stack step size was 1 µm (dendrites) or 0.5 µm (spines).

#### Dendritic tree and spine analysis

Dendritic arbors from confocal image stacks (1-µm step size) were manually traced and later quantified by Sholl analysis as described above. Prior to spine analyses, high-magnification confocal image stacks (0.5-µm step sizes) were blindly deconvolved. Subsequent analysis of spine morphology and density was conducted using Imaris software (Bitplane, AG, Zurich, Switzerland). Spine values longer than 0.5 µm were considered to represent real spines. The ratio of the spine head diameter and spine minimum diameter (SHD/SMinD) was used for classification into different spine types as follows: Stubby: SHD/SMinD < 1.5, length < 1 μm; Mushroom: SHD/SMinD > = 1.5 μm; Thin: 1 < SHD/SMinD < 1.5, length > 1 μm.

### Fluorescence recovery after photobleaching (FRAP)

Cultured hippocampal neurons from *Mkln1*^*–/–*^ and *Mkln1*^+/+^ mice were transfected with Actin-GFP Plasmid at DIV 12 and imaged the following day (DIV 13). Only mature spines with a distinct head were selected for analysis. Image acquisition was performed using a Nikon spinning disc confocal microscope (Visitron, Puchheim, Germany) equipped with ×60 objectives, 405/488 nm lasers, and an incubation chamber (5% CO_2_, 37 °C). Each spine was imaged five times (1 s per frame) using 488 nm excitation before photobleaching. On the sixth frame, photobleaching (total bleaching time of 1 s) was induced with ∼2.2 mW of laser power (405 nm laser). Imaging resumed immediately after bleaching and continued every second for 300 consecutive seconds. The recovery of the bleached fluorescence signal for each frame was normalized to the background and pre-bleach signals, as previously described^92^. The recovery curves were fitted to an exponential equation^92^ to extract various measures, including the relative size of the stable and dynamic pools and recovery half-time (t_1/2_).

### Preparation of synaptosome-enriched fractions and western blotting

#### Preparation of synaptosomes

Mice from both genotype groups were euthanized, and brains were immediately extracted. Hippocampi were isolated and stored in 10 volumes of sucrose buffer 1 (320 mM sucrose, 1 mM NaHCO_3_, 1 mM MgCl_2_, 0.5 mM CaCl_2_, 1 µM PMSF) containing protease inhibitor cocktail (Roche, Mannheim, Germany). Tissues were homogenized for 10 strokes using a motor-driven 2 ml Potter-Elvehjem homogenizer fitted with a Teflon pestle. All procedures were conducted at 4 °C with pre-cooled solutions. Homogenates were centrifuged at 1000 × *g* for 10 min. The resulting supernatant were stored on ice while pellets were re-homogenized once more and centrifuged at 700 × *g* for 10 min. The resulting supernatants were combined with the first supernatant and centrifuged at 13,800×*g* for 10 min. Pellets were homogenized in 500 µl sucrose buffer 2 (320 mM sucrose, 1 mM NaHCO_3_). Homogenates were overlaid with 2 ml of 1.4 M sucrose, 1 mM NaHCO_3_, and 2 ml of 1.0 M sucrose, 1 mM NaHCO_3_, followed by gradient centrifugation at 82,500 × *g* for 90 min. Bands at the 1.4 M and 1 M interface were collected, diluted in 4 volumes of sucrose buffer 2, and pelleted by centrifugation at 28,000 × *g* for 20 min. The resulting pellets containing synaptosomes were resuspended in 100 µl sucrose buffer 2. An equal volume of 1% (v/v) Triton X-100, 320 mM sucrose, 12 mM Tris-HCl pH 8.0 was added for 15 min with occasional mixing. Samples were centrifuged at 70,000 × *g* for 1 h. The resulting supernatant was kept as an insoluble fraction. The resulting pellet was resuspended in 40 mM Tris-HCl pH 8.0 and kept as an insoluble fraction. Sample protein concentrations were determined used bicinchoninic acid (BCA) assay (Pierce Biotechnology, Rockford, IL). Equal amounts of protein samples were applied on 4–15% gradient sodium dodecyl sulfate-polyacrylamide (SDS) gels.

#### Antibodies

The following antibodies were used for western blotting: mouse anti-Synaptotagmin (1:250; Abcam, Cambridge, UK); rabbit anti-GluA1 (1:1000, Thermo Fisher Scientific, Dreieich, Germany); mouse anti-GluA2 (1:1000; Millipore, Schwalbach, Germany); mouse anti-NR1 (1:1000; BD Biosciences, San Jose, CA); rabbit anti-NR2A (1:1000; Abcam, Cambridge, UK); mouse anti-NR2B (1:1000; Abcam, Cambridge, UK); mouse anti-PSD-95 (1:1000; Thermo Fisher Scientific, Dreieich, Germany); mouse anti-β-actin (1:5000; Sigma, Taufkirchen, Germany); mouse anti-GAPDH (1:5000; GeneTex, Irvine, CA), peroxidase-conjugated goat anti-mouse and goat anti-rabbit (1:15,000; Dianova, Hamburg, Germany).

#### Western blotting

Protein samples from presynaptic (10 µg) and postsynaptic (5 µg) fractions were subjected to sodium dodecyl sulfate-polyacrylamide gel electrophoresis (SDS-PAGE). Resolved bands were transferred to polyvinylidene difluoride (PVDF) membranes using a semi-dry blotting system. Membranes were first blocked in 5% bovine serum albumin (BSA) and incubated in primary antibodies for 1 h (GAPDH, β-actin), 2 h (GluA2, NR1) at RT, or overnight at 4 °C. The membranes were rinsed prior to incubation with horseradish peroxidase-conjugated secondary antibodies. Signals were detected with the SuperSignal WestPico Chemiluminescent Substrate Kit (Fisher Scientific). Signal intensities were analyzed using Image J (NIH) and normalized to Glyceraldehyde 3-phosphate dehydrogenase (GAPDH) loading controls. *Mkln1*^+/+^ control signal intensities were set to 100%.

### Statistics and reproducibility

Statistical analyses were conducted using SPSS (Version 26.0, Armonk, NY, IBM Corp.) and GraphPad Prism (Versions 6.0 and 9.0; GraphPad Software, Inc., San Diego, California, USA). All data were first investigated for deviation from a normal distribution with the Shapiro–Wilk’s *W* statistic and homogeneity of variances using Levene’s test. Behavioral data were subjected to analysis of variance (ANOVA) with independent factors of genotype (*Mkln1*^*–/–*^ vs. *Mkln1*^+/+^) and sex (male vs. female). Data extracted from repeated testing were subjected to mixed-design ANOVA with between-subject (genotype and sex) factors and the inclusion of time bins, days, or stimulus type as within-subject factors. Restricted analyses and Bonferroni corrected pairwise comparisons were performed to assist in data interpretation following significant interaction terms. Data from the social interaction paradigm and water maze probe test were also analyzed using a one-sample *t*-test, comparing against chance values as indicated in the figure legends. When no significant genotype × sex interaction terms emerged from analyses of the dependent variables, data for both sexes were pooled for illustrative purposes to avoid redundant graphs as the phenotype in both male and female mice was essentially in the same direction. The statistical outcomes are reported in Supplementary Table 1 and the corresponding figure legends.

For non-behavioral data, the Student’s *t*-test (or Student’s *t*-test with Welch’s correction for unequal variances) was used to compare two normally distributed independent sample groups. The Mann–Whitney *U* test was used to compare data from two independent samples (genotype) when data failed to fulfill the assumptions of parametric analyses. The two-sample Kolmogorov–Smirnov (KS) test was used to compare the distribution of interevent intervals, current amplitudes, and spine morphology characteristics. Dendritic branching was examined using repeated measures (radius) ANOVA and Bonferroni corrected pairwise comparisons following significant interaction terms. Data from spine density and FRAP analysis was subjected to linear mixed modeling with fixed (genotype) and random (e.g., neuron ID, Prep ID) effects. The comparison of nested models (with or without random effects) was conducted by testing the 2-log likelihood of the Chi-square distribution on a smaller-is-better basis. The final analysis for each specific parameter was based on the significantly best model, including both fixed and random effects. Fixed-effect parameter estimates are reported. Data yielding statistical values with an associated probability of *P* < 0.05 were interpreted as statistically significant.

### Reporting summary

Further information on research design is available in the Nature Research Reporting Summary linked to this article.

## Supplementary information

Supplementary Information

Description of Additional Supplementary Files

Supplementary Data 1

Supplementary Data 2

Supplementary Data 3

Supplementary Data 4

Supplementary Data 5

Supplementary Data 6

Supplementary Data 7

NR Reporting Summary

## Data Availability

Original un-cropped western blot scans are provided in Supplementary Figs. 5 and 6. Source data for the graphs and matrices in the main and Supplementary figures are provided in Supplementary Data 1 to 7.
