# Supplementary material for: Muskelin regulates actin-dependent synaptic changes and intrinsic brain activity relevant to behavioral and cognitive processes
Source: Commun Biol. 2022 Jun 15;5:589. doi: 10.1038/s42003-022-03446-1 (PMC9200775; doi:10.1038/s42003-022-03446-1)
Supplement: Supplementary file 1 — Supplementary Information [file 42003_2022_3446_MOESM1_ESM.pdf]

Supplementary Information

Muskelin regulates actin-dependent synaptic changes and intrinsic brain activity relevant to behavioral and cognitive processes

Mary Muhia, PingAn Yuanxiang, Jan Sedlacik, Jürgen R. Schwarz, Frank F. Heisler, Kira V. Gromova, Edda Thies, Petra Breiden, Yvonne Pechmann, Michael R. Kreutz, Matthias Kneussel

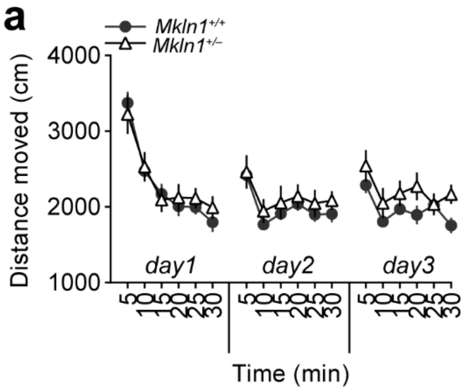

Supplementary Figure 1: Assessment of heterozygous animals in the open field test. (**a**) The assay shows comparable locomotor activity between heterozygous (*Mkln1*$^{+/-}$) and control (*Mkln1+/+*) mice. Distance moved is expressed in 5-min time bins across three days of testing. genotype: F (1,24) = 0.83, p = 0.37; genotype × day × time bins: F (10,240) = 0.623, p = 0.794). Data are presented as means ± SEM. *Mkln1*$^{+/+}$(N=13)*;* *Mkln1*$^{+/–}$(N=13).

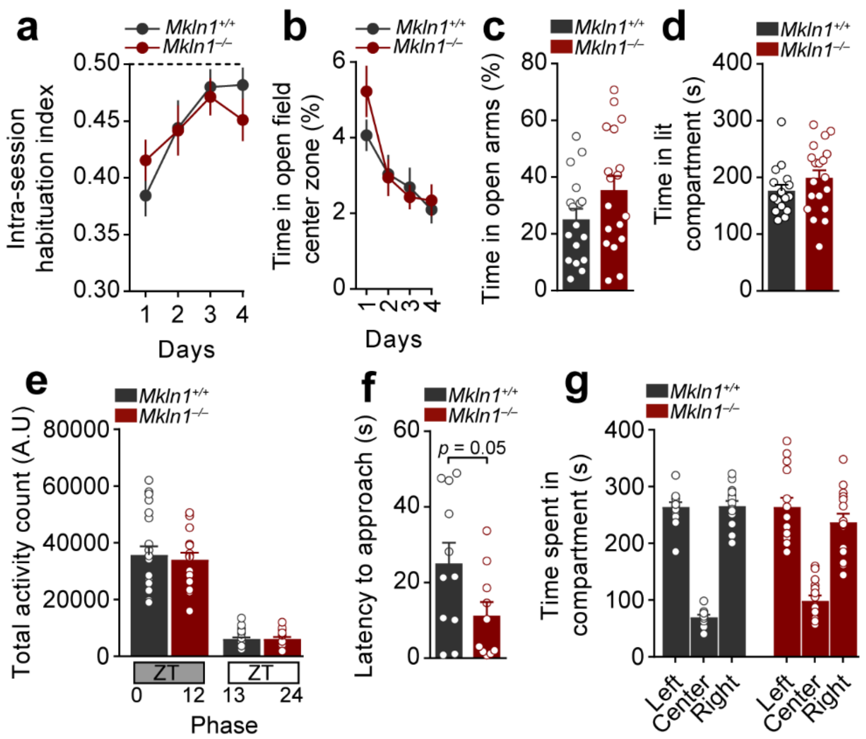

Supplementary Figure 2: *Mkln1-null* mice do not exhibit changes in anxiety-related behavior and home-cage activity (**a**) In the open field test, an intra-session activity change ratio reveals comparable within-session locomotor habituation between *Mkln1*$^{-/-}$ mice and *Mkln1*$^{+/+}$ controls. (**b-d**) Anxiety-related behavior is unaltered in *Mkln1*$^{-/-}$ mice. **(b)** Percentage time spent in the central zone of the open field arena on each day of testing is similar for *Mkln1*$^{-/-}$ and *Mkln1*$^{+/+}$ mice. (**c**) No genotype differences were detected in the proportion of time spent in the open arms of the elevated plus-maze during a 5-min test session. (**d**) Time spent in the open, brightly lit compartment of the light-dark transition test was comparable for both genotypes. (**e**) Bar graph showing total activity levels during the day (dark phase; ZT0-ZT12) and night (light phase; ZT13-ZT24) over three days of observation in the home cage. *Mkln1*$^{+/+}$ and *Mkln1*$^{-/-}$ mice show comparable levels of activity in both phases. (**f**) In the open field-plus-object test, the mean latency to the first contact with the object indicates a marginally significant tendency for decreased risk-taking or increased novelty-seeking behavior in *Mkln1*$^{-/-}$ mice (genotype: $F(1,17) = 4.35$, $p = 0.05$). (**g**) No differences were detected in the time spent in each compartment during the habituation phase of the 3-chambered social interaction paradigm. Scatter plots correspond to individual subjects. For data related to **a-b** (*Mkln1*$^{+/+}$(N=15: M = 8,

F = 7)); *Mkln1*$^{-/-}$(N=15: M = 8, F = 7); **c** (*Mkln1*$^{+/+}$ (N=17: M = 9, F = 8); *Mkln1*$^{-/-}$ (N=18: M = 11, F = 7); **d** (*Mkln1*$^{+/+}$ (N=19: M = 10, F = 9); *Mkln1*$^{-/-}$ (N=16: M = 9, F = 7); **e** (*Mkln1*$^{+/+}$ (N=22: M = 11, F = 11); *Mkln1*$^{-/-}$ (N=18: M = 11, F = 7);); **f** *Mkln1*$^{+/+}$ (N=11: M = 5, F = 6); *Mkln1*$^{-/-}$ (N=10: M = 5, F = 5); **g** (*Mkln1*$^{+/+}$ (N=15: M = 8, F = 7); *Mkln1*$^{-/-}$ (N=15: M = 8, F = 7). Data are presented as means ± SEM

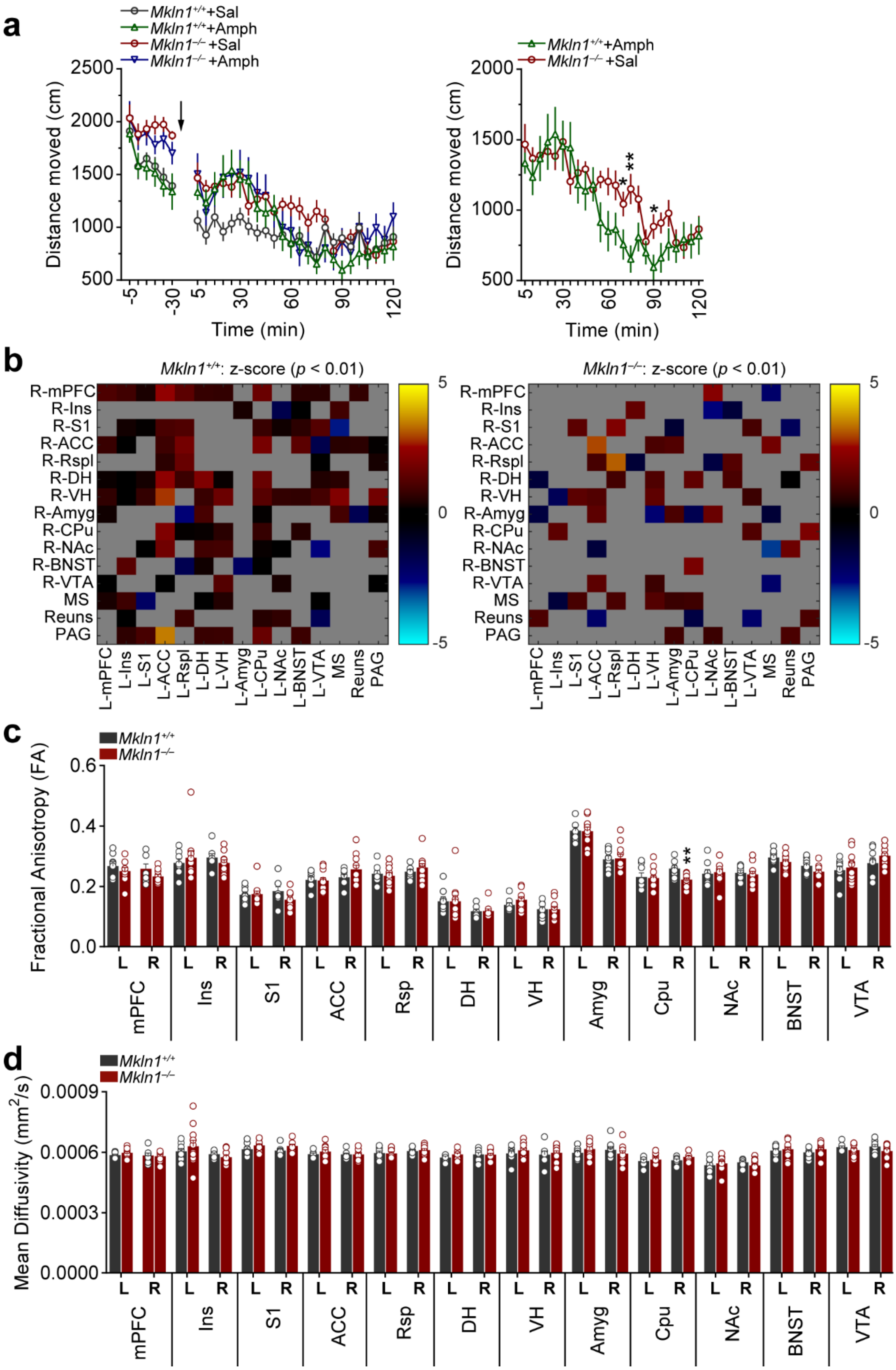
a
Mkln1+/++Sal
Mkln1+/++Amph
Mkln1−/− +Sal
Mkln1−/− +Amph
Distance moved (cm)
Time (min)
b
Mkln1+/+: z-score (p < 0.01)
Mkln1−/−: z-score (p < 0.01)
R-mPFC
R-Ins
R-S1
R-ACC
R-Rspl
R-DH
R-VH
R-Amyg
R-CPu
R-NAc
R-BNST
R-VTA
MS
Reuns
PAG
L-mPFC
L-Ins
L-S1
L-ACC
L-Rspl
L-DH
L-VH
L-Amyg
L-CPu
L-NAc
L-BNST
L-VTA
c
Fractional Anisotropy (FA)
mPFC
Ins
S1
ACC
Rsp
DH
VH
Amyg
Cpu
NAc
BNST
VTA
d
Mean Diffusivity (mm2/s)

Supplementary Figure 3: Reduced resting-state homotopic functional connectivity but largely intact brain diffusivity in *Mkln1-null* mice (**a**) Following a 30-min baseline recording session, *Mkln1*$^{+/+}$ and *Mkln1*$^{-/-}$ mice were injected with saline or 2.5 mg/kg amphetamine and assessed for 120 min. Baseline locomotion was significantly higher in *Mkln1*$^{+/+}$ mice compared with *Mkln1*$^{-/-}$ controls (genotype: $F(1,66) = 11.38$, $p < 0.01$). Arrow indicates the time of Amph administration. Without normalizing pre-drug differences in activity, saline-treated *Mkln1*$^{-/-}$ mice (right panel) show a similar locomotor profile as amphetamine-treated *Mkln1*$^{+/+}$ mice (genotype × time bins: $F(23,851) = 1.55$, $p < 0.05$ followed by Bonferroni post hoc test for pairwise comparisons (*$p < 0.05$, **$p < 0.01$)). Amph: *Mkln1*$^{+/+}$ (N=21: M = 10, F = 11); *Mkln1*$^{-/-}$ (N=18: M = 7, F = 11); Saline: *Mkln1*$^{+/+}$ (N=17: M = 8, F = 9); *Mkln1*$^{-/-}$ (N=18: M = 8, F = 10). (**b**) Correlation matrices depicting homotopic interhemispheric connectivity in *Mkln1*$^{+/+}$ (left panel) and *Mkln1*$^{-/-}$ (right panel) mice. Each element of the matrix represents the connectivity strength between homotopic regions. Interhemispheric *r* scores were transformed to *z* scores using Fisher's *r*-to-*z* transform. (**c-d**) Graphs illustrating the comparison of DTI-derived indices (Fractional anisotropy, FA (**b**) and Mean diffusivity, MD between *Mkln1*$^{+/+}$ and *Mkln1*$^{-/-}$ mice. Values represent mean ± SEM. **Abbreviations:** mPFC, medial prefrontal cortex; Ins, Insula; SS, somatosensory cortex; ACC, anterior cingulate cortex; Rspl, retrosplenial cortex; DH, dorsal hippocampus; VH, ventral hippocampus; Amyg, Amygdala; CPu, caudate-putamen; NAc, nucleus accumbens; MS, medial septum; BNST, bed nuclei of the stria terminalis; Reun, nucleus of reuniens; VTA, ventral tegmental area; PAG, Periaqueductal gray

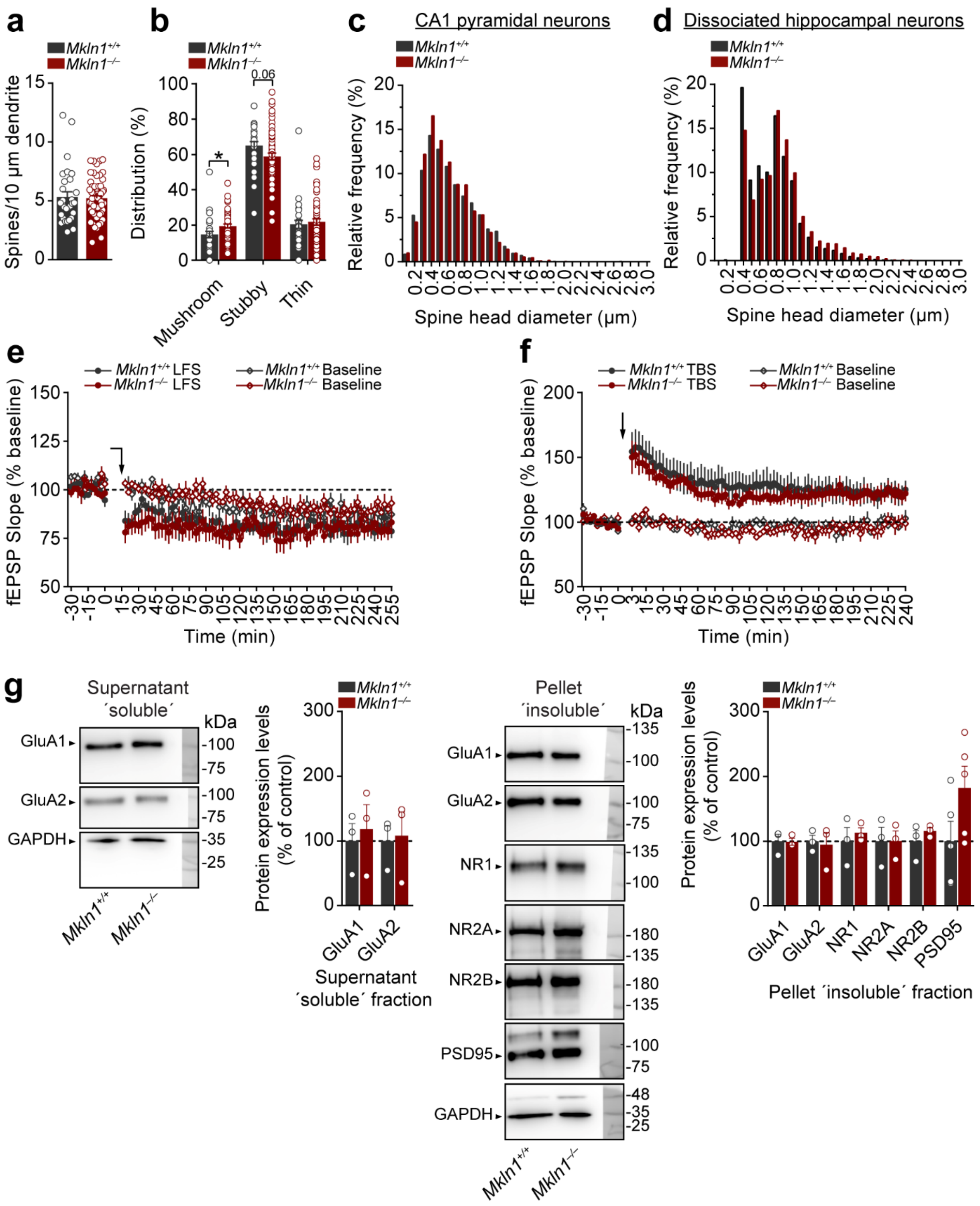

Supplementary Figure 4: Analysis of spine density and morphology and hippocampal plasticity in *Mkln1-null* mice. (**a**) Spine density is not altered in *Mkln1*$^{–/–}$ basal branches compared with *Mkln1*$^{+/+}$ branches (Genotype: F(1,87.07) = 0.074, p = 0.79). (**b**) Relative to *Mkln1*$^{+/+}$ control branches, analysis of spine types showed a significant increase in mushroom-

like spines (Genotype: $F(1,36.82) = 5.83$, $p < 0.05$) and decreased trend in stubby type spines ((Genotype: $F(1,89.99) = 3.74$, $p = 0.056$) in *Mkln1*$^{-/-}$ basal branches. (3 mice per genotype; *Mkln1*$^{+/+}$ (n = 11 neurons, 30 dendrites), *Mkln1*$^{-/-}$ (n = 20 neurons, 60 dendrites)). Data are shown as mean ± SEM. (**c**) Histogram showing the distribution of spine head diameters of all analyzed spines from *Mkln1*$^{-/-}$ and *Mkln1*$^{+/+}$ CA1 pyramidal neurons in 0.01 µm bins. (Two-sample KS test: $D = 0.044$, $p < 0.01$). (3 mice per genotype; *Mkln1*$^{+/+}$ (n = 67 dendrites, 2202 spines), *Mkln1*$^{-/-}$ (n =146 dendrites, 4592 spines)). (**d**) Histogram distribution of spine head diameters from dissociated hippocampal neurons of *Mkln1*$^{-/-}$ and *Mkln1*$^{+/+}$ mice (Two-sample KS test: $D = 0.095$, $p < 0.0001$). (5 independent preparations; *Mkln1*$^{+/+}$ (n = 55 neurons), *Mkln1*$^{-/-}$ (n =28 neurons). (**e**) Long-term depression (LTD) induced by low-frequency stimulation (LFS) of 900 pulses at 1Hz. Averaged fEPSP slope values normalized to 30 min baseline were comparable for slices in both genotype groups. Arrow indicates LFS from 0-15 min. Diamond traces illustrate baseline recordings. *Mkln1*$^{+/+}$ (N=3 mice); *Mkln1*$^{-/-}$ (N=4 mice). Baseline: *Mkln1*$^{+/+}$ (n=6 slices), *Mkln1*$^{-/-}$ (n=8 slices); LFS: *Mkln1*$^{+/+}$ (n=6 slices), *Mkln1*$^{-/-}$ (n=8 slices). (**f**) Long-term potentiation (LTP) at Schaffer collateral-CA1 synapses induced by five trains of theta-burst stimulation(4 pulses at 100 Hz, 200 ms between bursts). The averaged fEPSP slope normalized to 30 min baseline was comparable for *Mkln1*$^{-/-}$ and *Mkln1*$^{+/+}$ slices. Arrow indicates a 1s short-time stimulus. Diamond traces indicate baseline recordings. *Mkln1*$^{+/+}$ (N=4 mice); *Mkln1*$^{-/-}$ (N=4 mice). Baseline: *Mkln1*$^{+/+}$ (n=8 slices), *Mkln1*$^{-/-}$ (n=8 slices); LFS: *Mkln1*$^{+/+}$ (n=8 slices), *Mkln1*$^{-/-}$ (n=8 slices). Data are represented as mean ± SEM. (**g**) Analysis of PSD95 and receptor subunit levels in supernatant 'soluble' and pellet 'insoluble') fractionated extracts from hippocampal tissue. Mean values are reported as percentage of control and protein levels were normalized to the housekeeping gene, Glyceraldehyde 3-phosphate dehydrogenase (GAPDH). Protein expression levels in soluble and insoluble factions were comparable for *Mkln1*$^{+/+}$ and *Mkln1*$^{-/-}$ hippocampal tissue. *Mkln1*$^{+/+}$ (N=3-5)*;* *Mkln1*$^{-/-}$ (N=3-5).

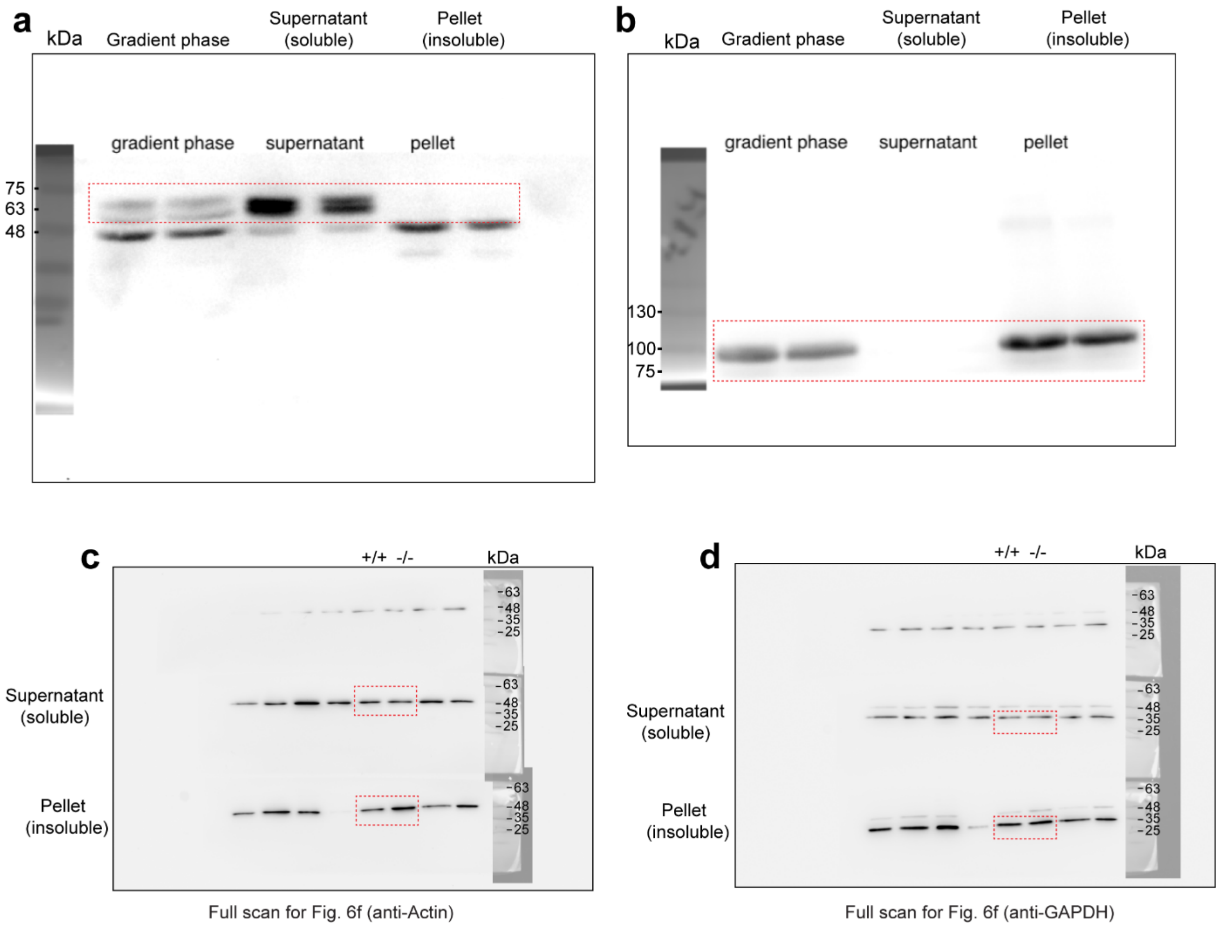

Supplementary Figure 5: Full-length scans of western blotting membranes. (**a-b**) Uncropped full-length scans of western blotting membranes presented in Fig. 6e. (**c-d**) Uncropped full-length scans of western blotting membranes presented in Fig. 6f. Areas outlined in dashed red square represent the cropped areas. Membranes were cut to allow blotting with different antibodies.

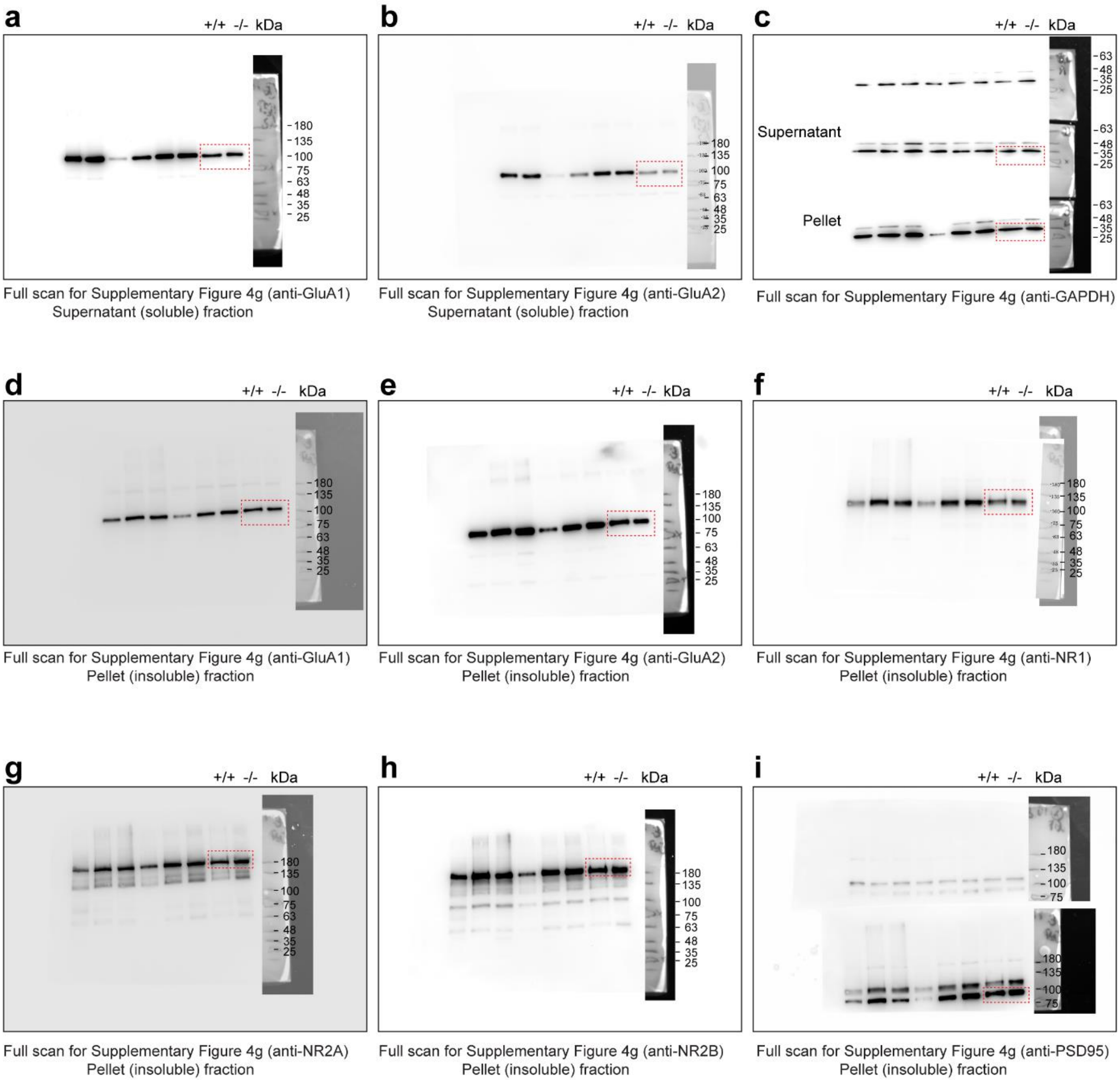

Supplementary Figure 6: Full-length scans of western blotting membranes. (**a-i**) Uncropped full-length scans of western blotting membranes presented in Supplementary Figure 4. Areas outlined in dashed red square represent the cropped areas. Membranes were cut to allow blotting with different antibodies. Membranes were stripped and reprobed with different antibodies.

Behavioral experiment	Figure	Measure	Genotype	Sex		Genotype*Sex	
			F (DFn, DFd); p-value	F (DFn, DFd); p-value	Mean values	F (DFn, DFd); p-value	Mean values
**Open field (4 days)**	Fig. 1a	Distance moved (cm)	F (1, 26) = 15.671; < 0.001***	F (1, 26) = 0.613; p = 0.441		F (1, 26) = 1.422; p = 0.244	
	Fig. 1b	Inter-session habituation index	F (1, 26) = 7.809; < 0.01**	**F (1, 26) = 8.823; p < 0.01****	Males (M)= 0.47 ± 0.009<br>Females (F) = 0.43 ± 0.01	F (1, 26) = 0.052; p = 0.822	
	Fig. S1 a	Intra-session habituation index	F (1, 26) = 0.015; p = 0.904	F (1, 26) = 1.953; p = 0.174		F (1, 26) = 0.688; p = 0.415	
	Fig. S1 b	Time in center zone (%)	F (1, 26) = 0.265; p = 0.611	F (1, 26) = 3.868; p = 0.060		F (1, 26) = 0.535; p = 0.471	
**Home cage activity**	Fig. 1c,<br>Fig. S1e	Total activity count (A.U)	F (1, 36) = 0.177; p = 0.676	**F (1, 36) = 10.599; p < 0.01****	Light: Males (M)= 5648.91 ± 574.57<br>Dark: Males (M) = 29295.27 ± 1952.07<br>Light: Females (F) = 6789.56 ± 715.29<br>Dark: Females (F) = 41959.44 ± 3072.93	**F (1, 36) = 9.737; p < 0.01****	*Mkln1* $^{+/+}$ (M)= Light (4832 ± 581.48); Dark (25005.82 ± 1862.35)<br>*Mkln1* $^{+/+}$ (F) = Light (7509.27 ± 917.78); Dark (46449.73 ± 3680.75)<br>*Mkln1* $^{-/-}$ (M) = Light (6465.82 ± 956.53); Dark (33584.73 ± 2975.93)<br>*Mkln1* $^{-/-}$ (F) = Light (5658.57 ± 1082.91); Dark (34903.29 ± 4442.58)
**Open field free exploration test**	Fig. 1f	Distance moved controlling for time spent in arena	F (1, 24) = 8.644; p < 0.01**	F (1, 24) = 0.025; p = 0.875		F (1, 24) = 0.230; p = 0.636	
**Open field-plus-object test**	Fig. 1h	Total distance moved (cm)	F (1, 17) = 2.924; p = 0.105	F (1, 17) = 0.041; p = 0.843		**F (1, 17) = 5.954; p < 0.05***	*Mkln1* $^{+/+}$ (M)= 18132.76 ± 1086.49<br>*Mkln1* $^{+/+}$ (F) = 23203.56 ± 2595.54<br>*Mkln1* $^{-/-}$ (M) = 26098.91 ± 1505.65<br>*Mkln1* $^{-/-}$ (F) = 21802.11 ± 1669.34
	Fig. 1i	Object exploration (sec)	F (1, 17) = 10.387; p < 0.01**	F (1, 17) = 1.719; p = 0.207		**F (1, 17) = 4.984; p < 0.05***	*Mkln1* $^{+/+}$ (M)= 33.68 ± 13.25<br>*Mkln1* $^{+/+}$ (F) = 58.75 ± 21.25<br>*Mkln1* $^{-/-}$ (M) = 182.11 ± 40.30<br>*Mkln1* $^{-/-}$ (F) = 85.70 ± 28.39
	Fig. S1 f	Latency to approach (sec)	F (1, 17) = 4.346; p = 0.052	F (1, 17) = 0.031; p = 0.861		F (1, 17) = 1.880; p = 0.188	
**3-Chamber Social interaction test**	Fig. 1j	Active exploration time (s)	F (1, 26) = 1.642; p = 0.211	F (1, 26) = 0.010; p = 0.921		F (1, 26) = 0.340; p = 0.565	
	Fig. 1k	Active exploration time (s)	F (1, 26) = 3.171; p = 0.087	F (1, 26) = 1.185; p = 0.286		F (1, 26) = 0.242; p = 0.627	
	Fig. 1m	Discrimination index	F (1, 26) = 8.245; p < 0.01**	F (1, 26) = 0.378; p = 0.544		F (1, 26) = 1.515; p = 0.229	
	Fig. S1 g	Time spent in compartments (s)	F (1, 26) = -7.184e -15; p = 1.000	F (1, 26) = -5.900e -15;p = 1.000		F (1, 26) = -9.866e -15;p = 1.000	
**Elevated Plus maze**	Fig. S1 c	Time in open arms (%)	F (1, 31) = 2.521; p = 0.122	F (1, 31) = 0.004; p = 0.947		F (1, 31) = 6.786e-4; p = 0.979	
**Light-Dark transition test**	Fig. S1 d	Time in lit compartment	F (1, 31) = 1.544; p = 0.223	F (1, 31) = 0.434; p = 0.515		F (1, 31) = 0.023; p = 0.879	
**Locomotor response to amphetamine**	Fig. 2a	%Change in locomotion	F (1, 66) = 1.044; p =0.311	F (1, 66) = 4.098; p < 0.05*	Saline: Males (M)= 60.60 ± 3.83<br>Amph: Males (M) = 72.18 ± 8.09<br>Saline: Females (F) = 67.23 ± 3.43<br>Amph: Females (F) = 83.37 ± 9.38	F (1, 66) = 0.006; p = 0.938	
	Fig. 2b	%Change in locomotion	F (1, 66) = 5.467; p < 0.05*	F (1, 66) = 2.042; p = 0.158		F (1, 66) = 0.005; p = 0.942	
**Acoustic startle response**	Fig. 2c	Startle amplitude (A.U)	F (1, 16) = 0.497; p = 0.491	F (1, 16) = 0.262; p = 0.616		F (1, 16) = 0.349; p = 0.563	
**Prepulse inhibition**	Fig.2d	Pulse-induced startle (A.U)	F (1, 26) = 1.642; p = 0.211	F (1, 26) = 3.248; p = 0.083		F (1, 26) = 0.965; p = 0.335	
	Fig.2e	Prepulse-induced startle (A.U)	F (1, 26) = 2.302; p = 0.141	F (1, 26) = 0.430; p = 0.518		F (1, 26) = 1.071e -5; p = 0.997	
	Fig.2f	Percent prepulse inhibition	F (1, 26) = 0.097; p = 0.758	F (1, 26) = 4.006; p = 0.056		F (1, 26) = 0.601; p = 0.445	
**Habituation of acoustic startle response**	Fig. 2g	Startle amplitude (A.U)	F (1, 17) = 1.439; p = 0.247	F (1, 17) = 0.543; p = 0.471		F (1, 17) = 0.840; p = 0.372	
**Water maze spatial reference memory**	Fig. 3a	Average swim speed (cm/s)	F (1, 26) = 24.907; p < 0.001**	F (1, 26) = 0.314; p = 0.580		F (1, 26) = 0.098; p = 0.756	
	Fig. 3b	Visible platform: Path length (cm)	F (1, 26) = 0.941; p = 0.341	F (1, 26) = 0.288; p = 0.596		F (1, 26) = 2.124; p = 0.157	
	Fig. 3b	Hidden platform: Path length (cm)	F (1, 26) = 6.973; p < 0.05*	F (1, 26) = 1.185; p = 0.286		F (1, 26) = 0.636; p = 0.432	
	Fig. 3d	Mean proximity to platform (cm)	F (1, 26) = 5.874; p < 0.05*	F (1, 26) = 3.547; p = 0.071		F (1, 26) = 3.020; p = 0.094	
**Cued fear conditioning and extinction**	Fig. 3f	Conditioned acqusition: %Time freezing	F (1, 34) = 0.157; p = 0.694	F (1, 34) = 1.065; p = 0.309		F (1, 34) = 0.008; p = 0.931	
	Fig. 3g	Extinction: %Time freezing	F (1, 34) = 5.270; p < 0.05*	F (1, 34) = 15.893; p < 0.001**	Males (M)= 15.79 ± 2.91<br>Females (F) = 28.69 ± 2.12	F (1, 34) = 1.477; p = 0.233	
	Fig. 3h	Spontaneous recovery: %Time freezing	F (1, 34) = 1.234; p = 0.274	F (1, 34) = 15.120; p < 0.001**	Males (M)= 26.98 ± 4.68<br>Females (F) = 47.30 ± 3.04	F (1, 34) = 1.399; p = 0.245	
	Fig. 3i	Extinction retention: %Time freezing	F (1, 34) = 5.293; p < 0.05*	F (1, 34) = 21.711; p < 0.001**	Males (M)= 11.05 ± 2.16<br>Females (F) = 26.47 ± 2.50	F (1, 34) = 0.546; p = 0.465	
	Fig. 3j	Fear renewal: %Time freezing	F (1, 34) = 4.536; p < 0.05*	F (1, 34) = 6.566; p < 0.05*	Males (M)= 18.48 ± 4.00<br>Females (F) = 31.21 ± 3.68	F (1, 34) = 3.800; p = 0.060	

Supplementary Table 1: Summary of statistical results for between-subject factors (genotype vs. sex) for the behavioral experiments. Values highlighted in grey are also detailed in the figure legends together with significant within-subject interaction terms.
