# Supplementary material for: Muskelin regulates actin-dependent synaptic changes and intrinsic brain activity relevant to behavioral and cognitive processes
Source: Commun Biol. 2022 Jun 15;5:589. doi: 10.1038/s42003-022-03446-1 (PMC9200775; doi:10.1038/s42003-022-03446-1)
Supplement: Supplementary file 2 — Description of Additional Supplementary Files [file 42003_2022_3446_MOESM2_ESM.pdf]

Description of Additional Supplementary Files

File name: Supplementary Data 1
Description: Data file related to Fig 1.

File name: Supplementary Data 2
Description: Data file related to Figs S1 and S2.

File name: Supplementary Data 3
Description: Data file related to Fig 2 and S3a.

File name: Supplementary Data 4
Description: Data file related to Fig 3.

File name: Supplementary Data 5
Description: Data related to Fig.4a-f and S3b-d.

File name: Supplementary Data 6
Description: Data file related to Fig 5 and S4a-d.

File name: Supplementary Data 7
Description: Data file related to Fig 6 and S4e-g.
