# Supplementary material for: Muskelin regulates actin-dependent synaptic changes and intrinsic brain activity relevant to behavioral and cognitive processes
Source: Commun Biol. 2022 Jun 15;5:589. doi: 10.1038/s42003-022-03446-1 (PMC9200775; doi:10.1038/s42003-022-03446-1)
Supplement: Supplementary file 10 — NR Reporting Summary [file 42003_2022_3446_MOESM10_ESM.pdf]

nature portfolio

Corresponding author(s): Matthias Kneussel, Mary Muhia

Last updated by author(s): Apr 12, 2022

Reporting Summary

Nature Portfolio wishes to improve the reproducibility of the work that we publish. This form provides structure for consistency and transparency in reporting. For further information on Nature Portfolio policies, see our Editorial Policies and the Editorial Policy Checklist.

Statistics

For all statistical analyses, confirm that the following items are present in the figure legend, table legend, main text, or Methods section.

n/a	Confirmed	
☐	☒	The exact sample size (n) for each experimental group/condition, given as a discrete number and unit of measurement
☐	☒	A statement on whether measurements were taken from distinct samples or whether the same sample was measured repeatedly
☐	☒	The statistical test(s) used AND whether they are one- or two-sided<br>*Only common tests should be described solely by name; describe more complex techniques in the Methods section.*
☐	☒	A description of all covariates tested
☐	☒	A description of any assumptions or corrections, such as tests of normality and adjustment for multiple comparisons
☐	☒	A full description of the statistical parameters including central tendency (e.g. means) or other basic estimates (e.g. regression coefficient) AND variation (e.g. standard deviation) or associated estimates of uncertainty (e.g. confidence intervals)
☐	☒	For null hypothesis testing, the test statistic (e.g. F, t, r) with confidence intervals, effect sizes, degrees of freedom and P value noted<br>*Give P values as exact values whenever suitable.*
☒	☐	For Bayesian analysis, information on the choice of priors and Markov chain Monte Carlo settings
☐	☒	For hierarchical and complex designs, identification of the appropriate level for tests and full reporting of outcomes
☒	☐	Estimates of effect sizes (e.g. Cohen's d, Pearson's r), indicating how they were calculated

Our web collection on statistics for biologists contains articles on many of the points above.

Software and code

Policy information about availability of computer code

Data collection	Noldus Ethovision XT 8.5, Startle Reflex Lab (SAR-LAB) software, Med Associates Video Freeze software, Olympus FluoView software, PWIN software (LIN, Magdeburg), Pathmaster software, Fitmaster, Igor Pro 6.03, Mini Analysis
Data analysis	Data were analyzed and plotted using Autoquant Deconvolution software, FIJI Image J, Excel, Graphpad Prism versions 5 and 7, Matlab, and IBM SPSS Statistics. Figures were assembled using Adobe Illustrator

For manuscripts utilizing custom algorithms or software that are central to the research but not yet described in published literature, software must be made available to editors and reviewers. We strongly encourage code deposition in a community repository (e.g. GitHub). See the Nature Portfolio guidelines for submitting code & software for further information.

Data

Policy information about availability of data

All manuscripts must include a data availability statement. This statement should provide the following information, where applicable:

- Accession codes, unique identifiers, or web links for publicly available datasets
- A description of any restrictions on data availability
- For clinical datasets or third party data, please ensure that the statement adheres to our policy

Original un-cropped western blot scans are provided in Supplementary Figures 5 and 6. Source data for the graphs and matrices in the main and Supplementary figures are provided in Supplementary Data 1 to 7.

Field-specific reporting

Please select the one below that is the best fit for your research. If you are not sure, read the appropriate sections before making your selection.

☒ Life sciences ☐ Behavioural & social sciences ☐ Ecological, evolutionary & environmental sciences

For a reference copy of the document with all sections, see nature.com/documents/nr-reporting-summary-flat.pdf

Life sciences study design

All studies must disclose on these points even when the disclosure is negative.

Sample size	No statistical methods were used to predetermine sample sizes. In vivo experiments were conducted in adherence to the 3R principle, and numbers in each genotype group were based on previously published studies of similar paradigms.
Data exclusions	Following post-experiment validation of genotype identity, mice were excluded from the study if they were the wrong genotype (e.g., heterozygotes).
Replication	For in vivo studies, independent animals were used as replicates. Behavioral phenomena (e.g., anxiety or activity) were replicated using independent groups and using different paradigms or variations of the same paradigm. Non-behavioral data were replicated at least three times, which included biological replicates. All attempts at replication of included data were successful.
Randomization	Animals for drug experiments were randomly assigned to receive either saline or drug injection.
Blinding	Behavioral studies were performed by an experimenter blinded to genotype during testing. All in vivo measurements were performed using automated systems and tracking software. Non-behavioral data were conducted in a blinded fashion.

Reporting for specific materials, systems and methods

We require information from authors about some types of materials, experimental systems and methods used in many studies. Here, indicate whether each material, system or method listed is relevant to your study. If you are not sure if a list item applies to your research, read the appropriate section before selecting a response.

Materials & experimental systems

n/a	Involved in the study	
☐	☒	Antibodies
☐	☒	Eukaryotic cell lines
☒	☐	Palaeontology and archaeology
☐	☒	Animals and other organisms
☒	☐	Human research participants
☒	☐	Clinical data
☒	☐	Dual use research of concern

Methods

n/a	Involved in the study	
☒	☐	ChIP-seq
☒	☐	Flow cytometry
☐	☒	MRI-based neuroimaging

Antibodies

Antibodies used	The following antibodies were used for Western Blotting: mouse anti-Synaptotagmin (1:250; Abcam, Cambridge, UK); rabbit anti-GluA1 (1:1000, Thermo Fisher Scientific, Dreieich, Germany); mouse anti-GluA2 (1:1000; Millipore, Schwalbach, Germany); mouse anti-NR1 (1:1000; BD Biosciences, San Jose, CA); rabbit anti-NR2A (1:1000; Abcam, Cambridge, UK); mouse anti-NR2B (1:1000; Abcam, Cambridge, UK); mouse anti-PSD95 (1:1000; Thermo Fisher Scientific, Dreieich, Germany); mouse anti-β-actin (1:5000; Sigma, Taufkirchen, Germany); mouse anti-GAPDH (1:5000; GeneTex, Irvine, CA), peroxidase-conjugated goat anti-mouse and goat anti-rabbit (1:15000; Dianova, Hamburg, Germany). DAPI (1:1,000).<br>A description of all antibodies used in this study with their source and working dilutions is also detailed within the manuscript in the Materials and Methods section.
Validation	All primary and secondary antibodies used in the study are commercially available and validation procedures are stated in the manufacturer's website.

Eukaryotic cell lines

Policy information about cell lines

Cell line source(s)	Mouse primary neuron

Authentication	Neurons were freshly prepared in our laboratory from hippocampi of muskelin homozygous knockout (Mkln1–/–) mice and wild-type (Mkln1+/+) littermates maintained on the C57BL6 background.
Mycoplasma contamination	Neurons were not tested for mycoplasma contamination, however animals used to prepare the primary cultures were tested and were mycoplasma free.
Commonly misidentified lines (See ICLAC register)	None

Animals and other organisms

Policy information about studies involving animals; ARRIVE guidelines recommended for reporting animal research

Laboratory animals	Muskelin homozygous knockout (Mkln1–/–) mice and wild-type (Mkln1+/+) littermates of both sexes were used in this study. The Mkln1–/– mouse line was backcrossed seven generations into the C57BL/6 background before testing. Animals were approximately 12 weeks at the beginning of testing.
Wild animals	Not applicable
Field-collected samples	Not applicable
Ethics oversight	All animal studies complied with the European Communities Council Directive (2010/63/EU) on the protection of experimental animals and guidelines set forth by the German Animal Welfare Act. Experiments were conducted following approval by the ethics committee of the City of Hamburg (Behörde für Gesundheit und Verbraucherschutz, Fachbereich Veterinärwesen; No. 68/15 and No. 106/10).

Note that full information on the approval of the study protocol must also be provided in the manuscript.

Magnetic resonance imaging

Experimental design

Design type	Resting state
Design specifications	One scan per animal, 6:20min long scans
Behavioral performance measures	n/a

Acquisition

Imaging type(s)	Functional
Field strength	7T
Sequence & imaging parameters	gradient echo, EPI, FoV=20x20mm^2, matrix=64x64, slice thickness=0.3mm, axial orientation, TE=9ms, TR=2500ms, FA=90deg
Area of acquisition	whole brain

Diffusion MRI ☒ Used ☐ Not used

Parameters	12 directions, 2 averages, b=0 and 1000s/mm^2, no cardiac gating

Preprocessing

Preprocessing software	DTIFIT, FLIRT and FNIRT tools of the FMRIB software library (FSL) version 5.0<br>ttest2, partialcorr and Fisher r-to-z transformation of Matlab version 2018b
Normalization	Images were transformed non-linearly using anatomical b=0 images
Normalization template	Template was created by averaging over all animals, registration was repeated twice on the iteratively updated template
Noise and artifact removal	rsfMRI signals were corrected for linear drift and high-pass filtered (cut off frequency at 0.01 Hz)
Volume censoring	None

Statistical modeling & inference

Model type and settings	Independent t-test for comparing FA and MD values, partial correlation for rsfMRI analysis controlling for non-functional signal fluctuations of ROIs in WM and ventricles, rsfMRI group comparison using Fisher r-to-z transformation

Effect(s) tested	Differences in FA and MD and in rsfMRI ROI correlations between groups

Specify type of analysis: ☐ Whole brain ☒ ROI-based ☐ Both

Anatomical location(s)	ROIs were placed manually in anatomical b=0 template of all animals
Statistic type for inference (See Eklund et al. 2016)	n/a
Correction	n/a

Models & analysis

n/a	Involved in the study
☐	☒ Functional and/or effective connectivity
☒	☐ Graph analysis
☒	☐ Multivariate modeling or predictive analysis

Functional and/or effective connectivity	Partial correlation
